# The pathogenesis of therapy-related myeloid neoplasms from *TP53*-mutant clonal hematopoiesis

**DOI:** 10.1038/s41375-025-02839-5

**Published:** 2025-12-17

**Authors:** Jonas Fullin, Ebru Topçu, Karolina A. Zielińska, Roman R. Schimmer, Nancy Klemm, Christian Koch, Francisco Caiado, Melissa Lock, Cyril Doerdelmann, Marco M. Bühler, Joelle Tchinda, Kari J. Kurppa, Lubor Borsig, Philip H. Jones, Massimo Lopes, Markus G. Manz, Steffen Boettcher

**Affiliations:** 1 Department of Medical Oncology and Hematology, University Hospital Zurich and University of Zurich, Zurich, Switzerland; 2 Institute of Molecular Cancer Research, University of Zurich, Zurich, Switzerland; 3 Department of Pathology and Molecular Pathology, University Hospital Zurich, Zurich, Switzerland; 4 Laboratory for Oncology, Children’s Hospital Zurich, Zurich, Switzerland; 5 Institute of Biomedicine and Medicity Research Laboratories, University of Turku, Turku, Finland; 6 Department of Physiology, University of Zurich, Zurich, Switzerland; 7 Wellcome Sanger Institute, Hinxton, UK; 8 Department of Oncology, University of Cambridge, Hutchinson Research Centre, Cambridge Biomedical Campus, Cambridge, UK; 9 Comprehensive Cancer Center Zurich, Zurich, Switzerland

**Keywords:** Preclinical research, Acute myeloid leukaemia

## Abstract

Therapy-related acute myeloid leukemia and myelodysplastic neoplasms (t-AML/MDS) are devastating complications of chemo- or radiation therapy in patients treated for an unrelated primary malignancy. Cancer patients with *TP53*-mutant hematopoietic stem and progenitor cells (HSPCs) – a condition termed clonal hematopoiesis (CH) – are at a particularly high risk for t-AML/MDS. However, the pathogenesis of *TP53*-mutant t-AML/MDS, especially the role of the *TP53* allelic state (i.e., mono- vs. biallelic), and its prognostic impact in AML/MDS have remained only poorly understood. We developed novel in vitro and in vivo mouse models to investigate how mono- or biallelic *Trp53* mutations influence clonal expansion and leukemic progression from CH to t-AML/MDS. While HSPCs with monoallelic *Trp53* mutations gain clonal fitness but retain their genomic integrity under chemo- or radiation therapy, biallelic *Trp53* mutations result in genomic instability and are essential for leukemic transformation. Moreover, we provide proof of concept that non-mutational p53 inactivation, such as *MDM2* overexpression, can replicate the effects of biallelic *TP53* mutations, providing a possible explanation for cases of *TP53*-mutant AML/MDS that retain one wild-type *TP53* allele. Our findings elucidate the pathogenesis of *TP53*-mutant t-AML/MDS and support the classification of biallelic *TP53*-mutant AML/MDS as distinct clinical entities.

## Introduction

Clonal hematopoiesis (CH) is a pre-malignant condition of the blood-forming system, characterized by the expansion of somatic blood cell clones harboring mutations in leukemia driver genes [1–3]. CH is associated with an increased risk for hematological malignancies, including myelodysplastic neoplasms (MDS) and acute myeloid leukemia (AML) [1–5]. While most mutations commonly observed in CH confer only a low risk for AML development, mutations in *TP53* are strongly associated with progression from CH to AML [4, 5].

*TP53* (termed *Trp53* in mice) encodes for the tumor suppressor p53, which safeguards genomic integrity under cellular stress such as DNA damage and oncogenic hyperproliferation [6, 7]. It is the most frequently mutated gene in human cancer with, however, variable prevalence based on subtypes [6–8]. *TP53* mutations occur in 5% of de novo AML/MDS cases, but in more than 40% in therapy-related myeloid neoplasms (t-MNs), i.e., AML/MDS cases which emerge in patients after prior exposure to chemotherapy or radiation therapy for their primary cancer [9–12]. In both de novo AML/MDS and t-MNs, *TP53* mutations confer poor clinical outcomes due to resistance to standard chemotherapies, and high relapse rates [13–16].

Recently, the *TP53* allelic state – i.e., whether mutations are mono- or biallelic – has emerged as a critical yet controversial prognostic factor. A seminal study by Bernard *et al*. found that only biallelic *TP53* inactivation was strongly associated with well-known clinicogenomic features of *TP53*-mutant AML/MDS such as complex karyotypes, and poor outcomes [17]. However, later studies produced conflicting results [18–23]. These discrepancies have resulted in inconsistent incorporation of the *TP53* allelic state into clinicopathological classification systems. The 5th edition of the World Health Organization Classification recognizes biallelic *TP53* inactivation as a distinct category in MDS but not in AML [24], whereas the International Consensus Classification (ICC) considers *TP53* mutations in both, but ignores their allelic configuration [25].

Altogether, a better mechanistic understanding of the role of *TP53* and its allelic state during leukemogenesis is urgently needed. To this end, we developed novel murine in vitro and in vivo models of CH to assess the functional implications of monoallelic or biallelic *TP53* mutations under well-controlled experimental conditions of DNA damage, thereby mimicking chemo- or radiation therapy in cancer patients. We aimed to replicate the pathogenesis of t-MN arising from *TP53*-mutant CH, focusing on how *TP53* allelic states impact basic p53 function, clonal fitness, genomic stability, and leukemic transformation.

## Methods

### Cell line generation

ER-Hoxb8 cell lines were generated from bone marrow (BM) cells of *Trp53*-fl-R245W-GFP [26] mice as previously described [27] and a doxycycline-inducible Cre recombinase was introduced by lentiviral transduction. For experimental induction of the *Trp53*^R245W^ mutation, cells were treated with up to 2.5 μg/mL doxycycline for 48 h. For MDM2 overexpression studies, ER-Hoxb8 cells were additionally transduced with an SFFV-*Mdm2*-IRES-tRFP lentiviral vector.

### In vitro transformation assay

ER-Hoxb8 cells were subjected to three treatment cycles of 1-h exposure to 40 μM etoposide, followed by a 4-day drug-free recovery period. Subsequently, β-estradiol concentration in the culture media was halved every two days over two weeks and then removed. Cultures were monitored for sustained growth in β-estradiol-free conditions.

### In vivo studies

*Trp53*-fl-R245W-GFP;SCL-CreERT mice were bred to obtain homozygous or heterozygous *Trp53*-fl-R245W-GFP genotypes. To induce hematopoietic-specific *Trp53*^R245W^ mutations, 6–12 weeks old female mice received up to five daily intraperitoneal injections of 100 mg/kg tamoxifen. Five weeks later, they were either exposed to two sublethal γ-irradiation doses or left non-irradiated. Peripheral blood (PB) was collected every 2–4 weeks, and BM, spleen, and tumors were collected and analyzed.

## Results

### Generation of novel in vitro and in vivo models of *Trp53*-mutant clonal hematopoiesis

**Fig. 1:**
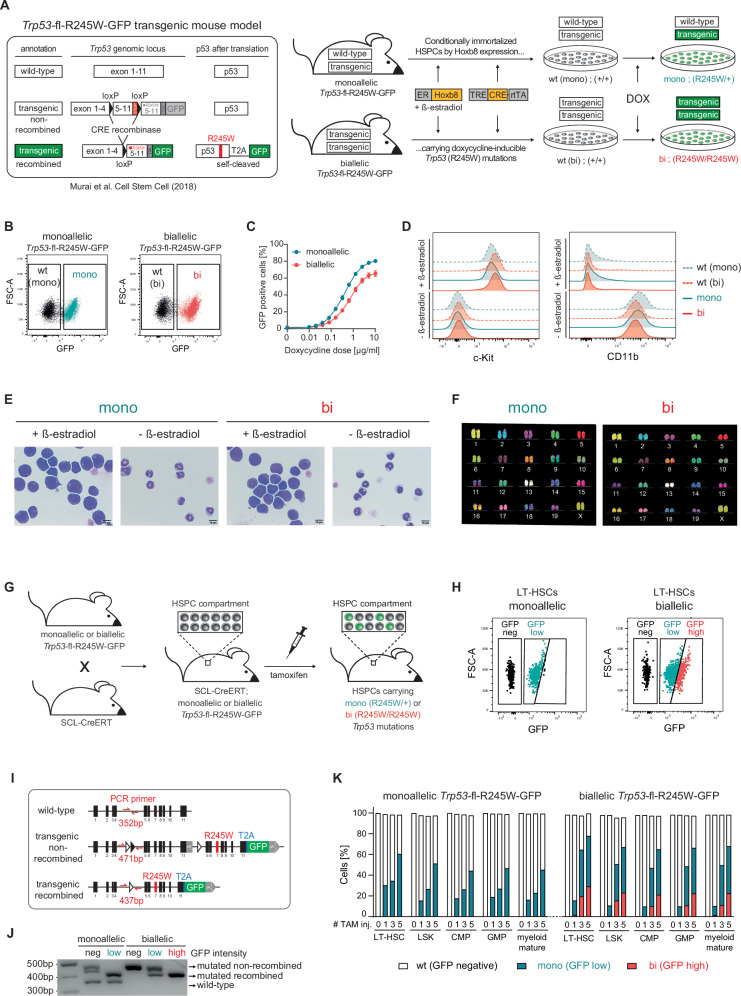
Generation and basic characterization of novel in vitro and in vivo model systems for *Trp53*-mutant clonal hematopoiesis. **A** Schematic representation of the workflow for generating ER-HoxB8 cell lines from *Trp53*-fl-R245W-GFP mice [26] with inducible mono- or biallelic *Trp53* mutations. **B** Cells were treated with 2.5 µg/mL doxycycline for 48 h and analyzed for GFP expression via flow cytometry; representative plots are shown. **C** GFP expression was measured by flow cytometry following treatment with the indicated doses of doxycycline for 48 h (biological replicates *n* = 3; symbols represent averages of experimental replicates, error bars indicate SEM) **D** Cells were cultured for 6 days in media with or without β-estradiol, stained for surface markers, and analyzed by flow cytometry (representative plots from *n* = 3 biological replicates shown). **E** Cytospins of ER-Hoxb8 cells cultured for 6 days in media with or without β-estradiol. Cells were stained with May-Grünwald-Giemsa solution, and imaged using light microscopy; representative images are shown. **F** Representative metaphase spreads of ER-Hoxb8 cells visualized with multicolor fluorescence in situ hybridization. **G** Schematic of the workflow for generating *Trp53-*fl-R245W-GFP;SCL-CreERT mice. **H** Mice were administered five daily doses of 100 mg/kg tamoxifen, and bone marrow (BM) cells were harvested 9 weeks post-treatment. Cells were stained for surface markers and analyzed by flow cytometry; representative GFP signals in long-term hematopoietic stem cell (LT-HSC) populations are shown. **I** Schematic illustrating locations of the PCR primers used to distinguish the allelic state of the *Trp53*-fl-R245W-GFP transgenes, as well as their Cre-mediated recombination status. **J** Agarose gel electrophoresis of PCR amplicons from DNA isolated from BM cells sorted into GFP^neg^, GFP^low^, and GFP^high^ populations. **K** Mice were injected with 100 mg/kg tamoxifen for the indicated number of times. After 9 weeks, BM cells were isolated, stained for surface markers and analyzed by flow cytometry to identify GFP expression in specific HSPC populations.

To establish in vitro models of *Trp53*-mutant CH, we isolated BM cells from mice carrying mono- or biallelic *Trp53*-fl-R245W-GFP alleles [26]. We conditionally immortalized these hematopoietic stem and progenitor cells (HSPCs) by estrogen receptor-driven homeobox B8 (ER-Hoxb8) expression [27], and introduced a doxycycline-inducible Cre recombinase. The resulting granulocyte-macrophage progenitor (GMP)-like [28] HSPC lines (Fig. 1A and Supplementary Fig. S1A) harbored the following features: (I) doxycycline-inducible and traceable switching from *Trp53*^WT^GFP^neg^ to *Trp53*^mutant^GFP^pos^ cells with either mono- (R245W/+) or biallelic (R245W/R245W) *Trp53* mutations (*Trp53*^WT(mono)^, *Trp53*^WT(bi)^, *Trp53*^mono^, or *Trp53*^bi^) (Fig. 1B), (II) titratable induction of mosaic mono- or biallelic *Trp53* mutations (Fig. 1C), (III) reversible immortalization upon β-estradiol removal leading to terminal myeloid differentiation (Fig. 1D, E and Supplementary Fig. S1B, C), and (IV) a normal karyotype (Fig. 1F).

In parallel, we generated an in vivo model by crossing *Trp53*-fl-R245W-GFP mice with the SCL-CreERT [29] strain, enabling tamoxifen-inducible, mosaic *Trp53* mutations in hematopoietic cells (Fig. 1G). Tamoxifen administration led to a single, uniformly GFP^pos^ population within the long-term hematopoietic stem cell (LT-HSC) compartment of mice with monoallelic *Trp53*-mutant alleles, while mice with biallelic *Trp53*-mutant alleles exhibited two GFP^pos^ populations with distinct intensities (Fig. 1H). PCR analysis from FACS-purified subpopulations confirmed that GFP^low^ cells had recombined a single *Trp53* allele, whereas GFP^high^ cells represent the truly biallelic state (Fig. 1I, J). Deep amplicon sequencing of GFP^neg^, GFP^low^, and GFP^high^ LSK cells provided supporting quantitative evidence (Supplementary Fig. S2A). This mouse model therefore allows for non-leaky and titratable induction of mutant *Trp53* in all HSPC and mature myeloid/lymphoid cells and enables direct comparison of all three *Trp53* allelic states in a single mouse, by comparing the subpopulations with distinct GFP intensities (Fig. 1K and Supplementary Figs. S2B, C, S3A).

### Allelic state-specific effects of mono- or biallelic *Trp53* mutations on p53 functionality and clonal fitness

**Fig. 2:**
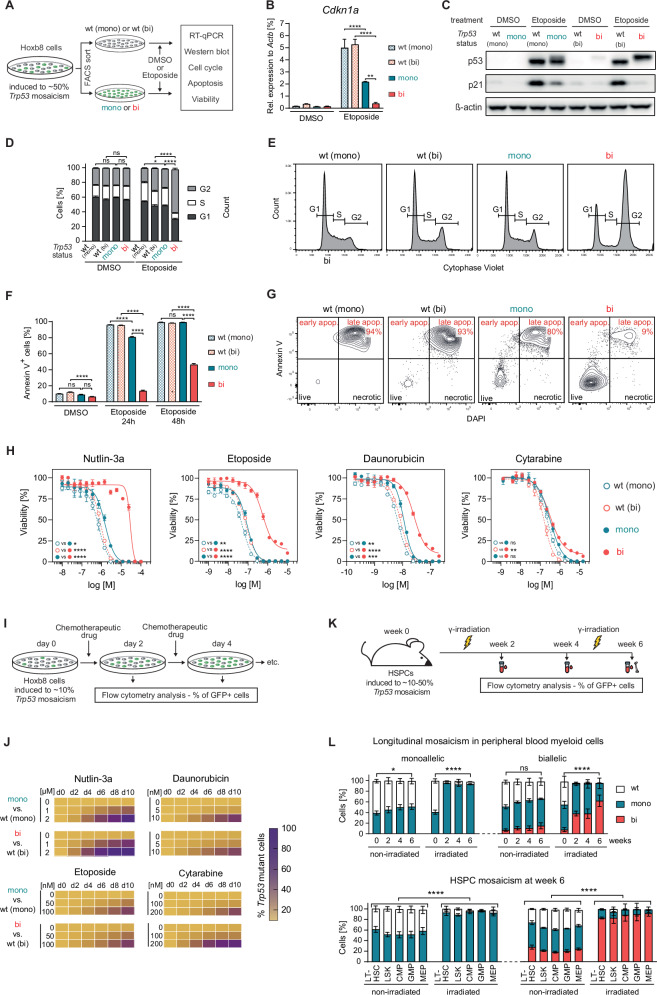
Impact of the *Trp53* allelic state on p53 functionality and clonal fitness. **A** Schematic representation of the workflow to assess p53 functionality in response to etoposide treatment. **B** Cells were treated with DMSO or 10 µM etoposide for 3 h. Total RNA was extracted, reverse-transcribed, and analyzed by qPCR to measure *Cdkn1a* transcript levels, normalized to *Actb* expression in the same samples (biological replicates *n* = 3; symbols represent averages of experimental replicates, error bars indicate SEM, *p < 0.05, ***p < 0.001,****p < 0.0001, one-way ANOVA, Tukey multiple comparison). **C** Cells were treated with either DMSO or 10 µM etoposide for 3 h. Whole-cell protein lysates were collected, separated on a polyacrylamide gel, and immunoblotted for p53, p21, and β-actin (biological replicates *n* = 3; representative image shown). **D** Cells were treated with DMSO or 40 µM etoposide for 1 h, followed by culture in drug-free medium for 24 h. Cells were then stained with CytoPhase Violet DNA-binding dye to assess cell cycle distribution (biological replicates *n* = 3; symbols represent the average of experimental replicates; error bars indicate SEM, *p < 0.05, ****p < 0.0001, one-way ANOVA, Tukey multiple comparison). **E** Gating strategy for cell cycle analysis using CytoPhase Violet. Representative plots of etoposide-treated samples are shown. **F** Cells were treated with DMSO or 1 µM etoposide for the indicated durations and subsequently stained with Annexin V. Flow cytometry was performed to evaluate the percentage of total apoptotic cells (biological replicates *n* = 3; symbols represent the average of experimental replicates; error bars indicate SEM, ***p < 0.001, ****p < 0.0001, one-way ANOVA, Tukey multiple comparison). **G** Gating strategy for the analysis of Annexin V positive cells. Combined frequencies of early and late apoptotic cells were used for plotting. Representative plots of etoposide-treated samples are shown. **H** Cells were treated with serial concentrations of the indicated drugs for 48 h, and cell viability was assessed using the CellTiter-Glo luminescent assay (biological replicates *n* = 3; symbols represent averages of experimental replicates, error bars indicate SEM, *p < 0.05, ***p < 0.001,****p < 0.0001, unpaired *t*-tests comparing the areas under the curve of the indicated comparisons). **I** Schematic of the experimental workflow for in vitro competitive cell growth assays. **J** Cells were induced to a mosaicism of 10% *Trp53* mutant cells using doxycycline. Cultures were exposed to the indicated drug concentrations for 10 days, and GFP^pos^ cells were quantified every 2 days via flow cytometry (biological replicates *n* = 3). **K** Schematic of the experimental workflow for in vivo competitive cell growth assays. **L** Hematopoietic cells of *Trp53*-fl-R245W-GFP;SCL-CreERT mice were induced to a mosaicism of 10–50% *Trp53* mutant cells, depending on the cell population, by tamoxifen administration. Mice were then divided into two groups: one group received two sublethal doses of γ-irradiation (475 cGy) 4 weeks apart, while the other remained non-irradiated. Peripheral blood and bone marrow cells were collected at the specified time points and analyzed for GFP expression in the indicated cell subtypes by flow cytometry (biological replicates *n* = 4; symbols represent averages of experimental replicates, error bars indicate SEM).

First, we assessed the impact of mono- or biallelic *Trp53* mutations on p53-mediated responses to DNA-damaging agents such as etoposide (Fig. 2A). Etoposide treatment led to increased p53 expression across all *Trp53* genotypes. In contrast, expression of the canonical p53 target p21 was strongly induced only in Hoxb8-*Trp53*^WT^ cells, while both its transcript (*Cdkn1a*) and protein levels were partially or fully reduced, in Hoxb8-*Trp53*^mono^ or Hoxb8-*Trp53*^bi^ cells, respectively (Fig. 2B, C). Consequently, Hoxb8-*Trp53*^bi^ cells failed to undergo a physiologic, p21-mediated G1 arrest and accumulated in G2, an effect less pronounced in Hoxb8-*Trp53*^mono^ cells (Fig. 2D, E). Similarly, Hoxb8-*Trp53*^mono^ cells exhibited only mild resistance to etoposide-induced apoptosis, whereas Hoxb8-*Trp53*^bi^ cells demonstrated a strong apoptotic defect (Fig. 2F, G). These defects led to a gradually increased resistance of Hoxb8-*Trp53*^mono^ or Hoxb8-*Trp53*^bi^ cells to multiple chemotherapeutic agents (Fig. 2H). Collectively, these results demonstrate allelic state-specific functional differences in canonical p53-regulated pathways between *Trp53*^mono^ or *Trp53*^bi^ HSPCs in vitro.

Given the low intrinsic fitness advantage of *TP53* mutations in CH [30, 31], we then evaluated the competitive fitness of *Trp53-*mutant Hoxb8 cells in the context of chemotherapy. Induction of ~10% mosaicism in Hoxb8-*Trp53*^mono^ or Hoxb8-*Trp53*^bi^ cells allowed us to compare the relative frequencies of *Trp53*^mutant^GFP^pos^ and *Trp53*^WT^GFP^neg^ populations over time (Fig. 2I). This revealed a strong and dose-dependent outgrowth of both, *Trp53*^mono^ and *Trp53*^bi^ cells over their *Trp53*^WT^ counterparts (Fig. 2J).

To examine *Trp53*-mutant clonal dynamics in vivo, we induced 10-50% PB cell mosaicism in SCL-CreERT*;Trp53-*fl-R245W-GFP mice and followed the various populations longitudinally (Fig. 2K, L). At steady-state, *Trp53*-mutant PB myeloid, B- and T-cell populations showed only minor expansion. However, following two sublethal γ-irradiation cycles, *Trp53*-mutant PB cells expanded significantly, with *Trp53*^bi^GFP^high^ cells strongly outcompeting *Trp53*^mono^GFP^low^ cells (Fig. 2L and Supplementary Fig. S4A). Terminal analysis revealed near-complete clonal dominance of *Trp53*^bi^ cells across all HSPC compartments in mice subjected to γ-irradiation. In summary, sublethal γ-irradiation drives clonal expansion of *Trp53*^mono^ over *Trp53*^WT^ HSPCs, while *Trp53*^bi^ HSPCs exhibit even greater clonal fitness.

### Biallelic but not monoallelic *Trp53* mutations result in genomic instability

**Fig. 3:**
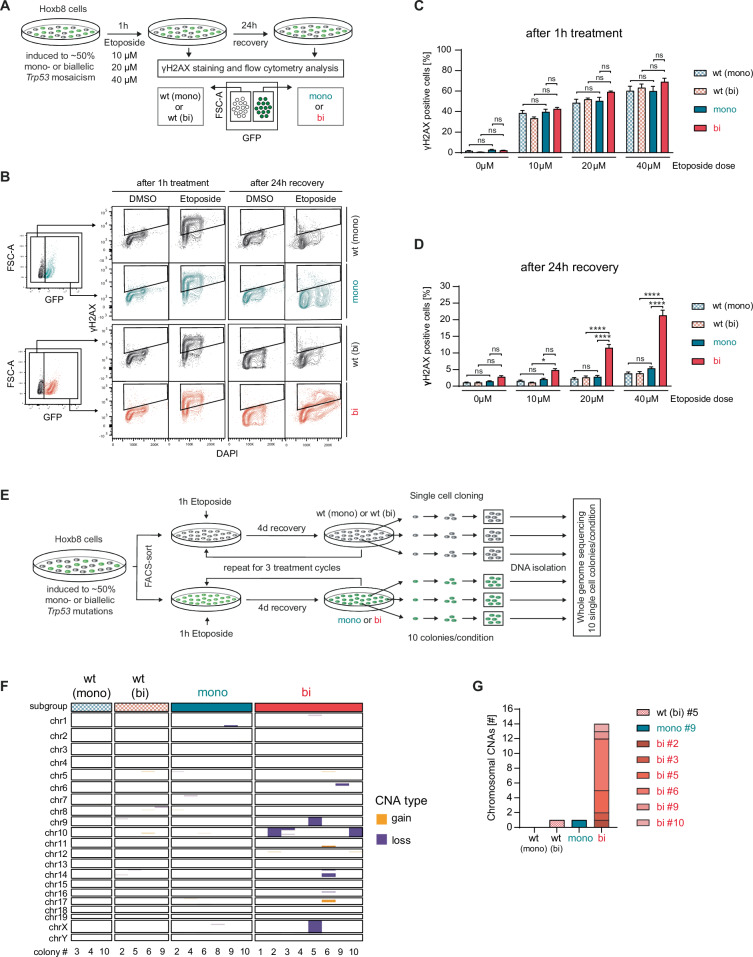
Biallelic *Trp53* mutations promote genomic instability. **A** Schematic of the experimental workflow for γH2AX analysis. **B** Gating strategy used for γH2AX analysis. **C**, **D** Cells were treated with the indicated doses of etoposide or DMSO for 1 h, followed by culture in drug-free medium for 24 h. Samples were collected immediately after the 1-h treatment and after the 24-h recovery period. Cells were fixed, permeabilized, and stained intracellularly with an antibody against γH2AX (biological replicates *n* = 3; symbols represent the average of experimental replicates; error bars indicate SEM, *p < 0.05, **p < 0.01, ***p < 0.001,****p < 0.0001, one-way ANOVA, Tukey multiple comparison). **E** Schematic representation of the experimental workflow for whole-genome sequencing of single-cell colonies. Cells were treated with DMSO or 40 µM etoposide for 1 h followed by culture in drug-free medium for 4 days. This treatment cycle was repeated for 3 times total, after which single cell colonies were grown. 10 colonies per genotype were subjected to whole genome sequencing. **F** Heatmap illustrating the size and nature of CNAs. All samples with detectable CNAs are displayed. **G** Quantification of chromosomal CNAs (copy number alterations defined with a size of >3 Mb). Different colors within each bar represent distinct single colonies derived from the same sample.

Given that complex karyotypic abnormalities are a hallmark of *TP53*-mutant myeloid malignancies [14], we examined whether the *Trp53* allelic state affects genomic integrity following DNA-damaging insults. We assessed γH2AX-positive cells, an indirect marker of DNA double strand breaks in Hoxb8-*Trp53*^mono^ or Hoxb8-*Trp53*^bi^ cells after etoposide treatment (Fig. 3A). After 1-hour etoposide exposure, γH2AX levels increased across all genotypes. However, after 24 h of recovery without etoposide, elevated γH2AX levels persisted specifically in Hoxb8-*Trp53*^bi^ cells (Fig. 3B–D). Since γH2AX is only a surrogate marker for DNA damage, we then investigated whether this increased persistence of DNA damage in *Trp53*^bi^ cells would translate into detectable genomic aberrations. To induce a high DNA damage load, we administered three consecutive 1-h etoposide treatment cycles, mimicking the chemotherapy treatment regimen in patients. Hereafter, we performed single-cell cloning and conducted whole-genome sequencing (WGS) on 10 individual clonal colonies per genotype (Fig. 3E). As expected from etoposide’s mechanism of action as topoisomerase II inhibitor, we found no increase in single nucleotide variants (SNVs) compared to untreated parental cell lines (Supplementary Fig. S4B). Thus, we focused on copy number alterations (CNAs). Among 30 sequenced colonies from *Trp53*^WT(mono)^, *Trp53*^WT(bi)^, and *Trp53*^mono^ cells, we detected only 2 CNAs >3000 kb – one CNA in one *Trp53*^WT(bi)^ and *Trp53*^mono^ colony each. By contrast, 6/10 *Trp53*^bi^ colonies harbored at least one large alteration, totaling 14 large-scale CNAs (Fig. 3F, G). These findings demonstrate that a single functional wild-type *Trp53* allele is sufficient to maintain genomic stability in HSPCs in vitro, whereas biallelic *Trp53* loss promotes the occurrence of large-scale CNAs.

### Biallelic *Trp53* mutations are essential for malignant transformation

**Fig. 4:**
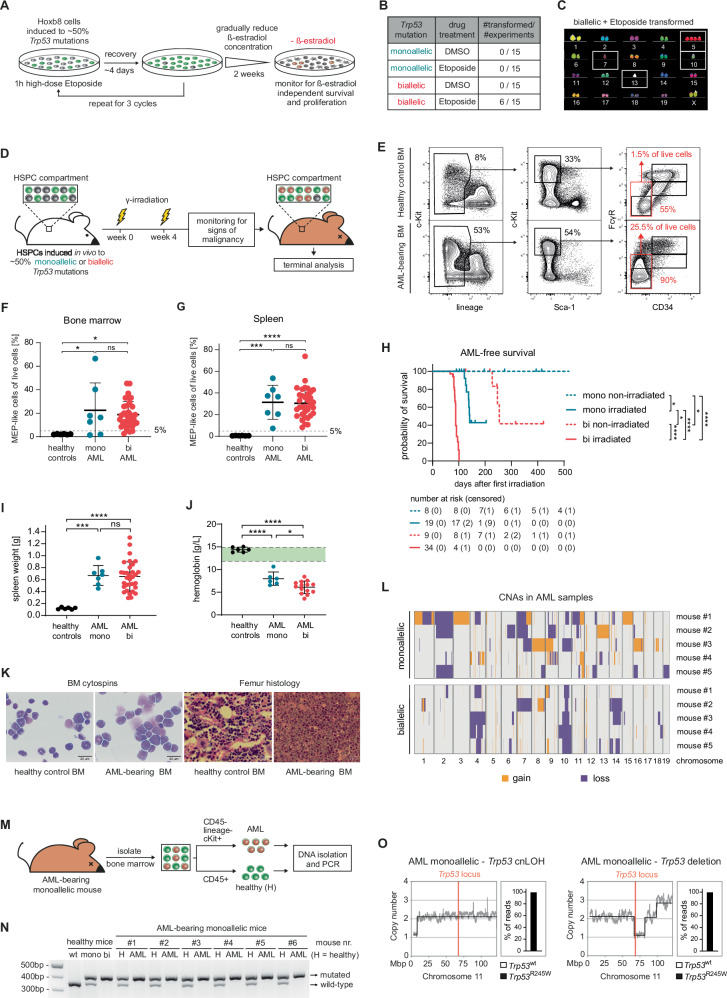
Biallelic *Trp53* mutations are essential for malignant transformation in vitro and AML development in vivo. **A** Schematic illustrating the experimental workflow to assess the transformation potential of ER-HoxB8 cells. Cells were treated with DMSO or 40 µM etoposide for 1 h, followed by culture in drug-free medium for 4 days. This treatment cycle was repeated three times, after which the β-estradiol concentration in the medium was gradually reduced over 2 weeks. Cultures were monitored for the emergence of cells capable of growing in the absence of β-estradiol. **B** Table summarizing the frequency of in vitro transformed cell line establishment across 15 independent experiments per condition. **C** Representative metaphase spread showing multicolor fluorescence in situ hybridization analysis of an in vitro transformed cell line. **D** Workflow for testing malignant transformation in *Trp53*-fl-R245W-GFP;SCL-CreERT mice. Mice were divided into two groups after induction of *Trp53* mutations: one received two sublethal doses of γ-irradiation (475 cGy) 4 weeks apart, while the other group remained non-irradiated. Mice were terminated upon showing signs of malignancy or after 500 days. Data shown are pooled from 5 independent experiments with total group sizes of *n* = 8, 9, 19, and 34 animals, respectively. **E** Representative immunophenotype of bone marrow cells from healthy and AML-bearing mice. **F** Frequency of MEP-like cells in the BM of AML-bearing and healthy control mice. **G** Frequency of MEP-like cells in the spleen of AML-bearing and healthy control mice. An MEP-like cell frequency of >5% in BM or spleen was used as a criterion for AML diagnosis. **H** Kaplan–Meier curves of AML-free survival, with non-AML-related deaths censored (*p < 0.05, ****p < 0.0001, Mantel–Cox test). **I** Spleen weights of healthy and AML-bearing mice. **J** Hemoglobin levels at the time of termination. **K** Representative cytomorphology of bone marrow cells and femoral histology sections. **L** Heatmap showing copy number alterations from whole-genome sequencing of purified AML cells, with healthy BM cells of the same mouse strain used as a reference. **M** Workflow illustrating the assessment of loss of heterozygosity at the *Trp53* locus using PCR. Bone marrow cells from AML-bearing mice with monoallelic *Trp53*-fl-R245W-GFP transgenes were sorted by FACS into AML blasts and healthy MEPs for DNA isolation and PCR analysis. **N** Agarose gel electrophoresis showing DNA amplicons from *Trp53*-specific PCR. **O** Copy number plots for chromosome 11 and *Trp53*^wt^ vs. *Trp53*^R245W^ read percentages to infer type of LOH in AMLs with monoallelic *Trp53*-fl-R245W-GFP transgenes. One representative example of copy-neutral loss of heterozygosity and *Trp53* deletion each is shown.

We next examined whether the increased genomic instability in biallelic *Trp53*-mutant cells enhances their potential for malignant transformation. To test this in vitro, we took advantage of the reversible immortalization of ER-Hoxb8 cells, which undergo terminal myeloid differentiation and ultimately die when β-estradiol is removed from the culture medium (Fig. 1D, E). This feature enabled identification of cells that had acquired etoposide-induced genomic aberrations leading to β-estradiol-independent enhanced self-renewal and differentiation arrest – two key hallmarks of leukemic transformation. After three etoposide cycles, we gradually reduced and eventually completely removed β-estradiol and monitored cultures for β-estradiol-independent cell growth (Fig. 4A). In 15 independent experiments, no β-estradiol-independent cell growth occurred in any DMSO-treated cells, or etoposide-treated *Trp53*^mono^ cells. However, in 6/15 experiments with *Trp53*^bi^ cells treated with etoposide, we observed emergence of continuously proliferating cells in the absence of β-estradiol (Fig. 4B). These in vitro transformed cells retained an immature immunophenotype and displayed myelodysplasia-like morphology (Supplementary Fig. S5A, B). Notably, multicolor in situ fluorescence hybridization analysis identified extensive chromosomal alterations, resembling the complex karyotypes commonly seen in t-MN patients with biallelic *TP53* mutations (Fig. 4C and Supplementary Fig. S5C).

To test the impact of the *Trp53* allelic state on the transformative potential of HSPCs in vivo, we exposed SCL-CreERT;*Trp53*-fl-R245W-GFP mice to two separated doses of sublethal γ-irradiation and monitored them for the occurrence of hematological malignancies (Fig. 4D). Overall survival was significantly reduced relative to the reported wild-type C57/BL6 life-span (676–878 days) [32, 33], with irradiated *Trp53*^bi^ mice exhibiting the shortest survival, followed by irradiated *Trp53*^mono^, non-irradiated *Trp53*^bi^, and non-irradiated *Trp53*^mono^ mice (Supplementary Fig. S6A). At time of euthanasia, virtually all mice had developed at least one type of hematologic malignancy, predominantly CD4^+^CD8^+^ thymic T cell lymphomas (Supplementary Fig. S6B–D). This was expected, as *Trp53*-mutant mouse models are well known to have a strong propensity for T cell lymphoma development [34, 35]. However, we additionally observed BM and spleen infiltration with immature, blast-like cells that resembled megakaryocyte-erythroid progenitors (MEPs) based on a Lin^-^c-Kit^+^Sca-1^-^CD34^-^FcγR^-^ immunophenotype (Fig. 4E). This population was present in almost all of the γ-irradiated *Trp53*^bi^ mice, in 30-40% of γ-irradiated *Trp53*^mono^, as well as non-irradiated *Trp53*^bi^ mice – but never in non-irradiated *Trp53*^mono^ mice (Supplementary Fig. S6B). These cells represent acute erythroid leukemia (AEL) – a subtype of AML almost uniformly associated with biallelic *TP53* mutations – further supported by loss of CD45 and high levels of CD71 (transferrin receptor) expression (Supplementary Fig. S6E). Since we aimed at studying the pathogenesis of therapy-related AML/MDS, we further focused on the characterization of these AML-bearing mice, as defined by the presence of an MEP-like blast population of >5% in BM and/or spleen (Fig. 4G, H). AML-free survival followed the same pattern as overall survival with the shortest latency to AML development in γ-irradiated mice with biallelic *Trp53* mutations followed by γ-irradiated mice with monoallelic *Trp53* mutations (Fig. 4H). Affected mice exhibited marked splenomegaly and severe anemia (Fig. 4I, J). BM cytomorphology and histology confirmed blast infiltration with disrupted BM architecture (Fig. 4K). The leukemic cells were transplantable and successfully engrafted in recipient mice, where they gave rise to AMLs with a comparable phenotype to the primary disease (Supplementary Fig. S7A–F). Furthermore, WGS analysis of AML cells revealed numerous CNAs, regardless of the initial *Trp53* allelic state of the mice (Fig. 4L). Given the identical disease phenotype but longer latency of AML development in γ-irradiated mice carrying monoallelic versus biallelic *Trp53* mutations, we wondered whether the AML blasts from mice with initially monoallelic *Trp53* mutations retained a wild-type allele. Therefore, we FACS-separated BM cells of AML-bearing mice with monoallelic *Trp53* mutations into pure AML (CD45^-^Lin^-^c-Kit^+^) and remaining healthy (CD45^+^) populations to identify wild-type and mutant alleles by PCR (Fig. 4M). Strikingly, while the healthy BM population expectedly showed both wild-type and mutant *Trp53* alleles, AML cells consistently lacked the wild-type allele (Fig. 4N). We confirmed this loss of heterozygosity (LOH) by WGS, indicating that copy number-neutral LOH or deletion of the chromosomal region encompassing the *Trp53* locus, accounted for the loss of the remaining wild-type allele (Fig. 4O).

Collectively, our data demonstrate that biallelic *Trp53* mutations are essential for malignant transformation of HSPCs in vitro as well as the in vivo development of highly aggressive AMLs – in particular of an erythroid phenotype, that is known to be associated with biallelic *TP53* mutations in AML patients [36, 37].

### Transcriptional consequences of mono- or biallelic *Trp53* mutations in pre-malignant HSPCs and in AML blasts

**Fig. 5:**
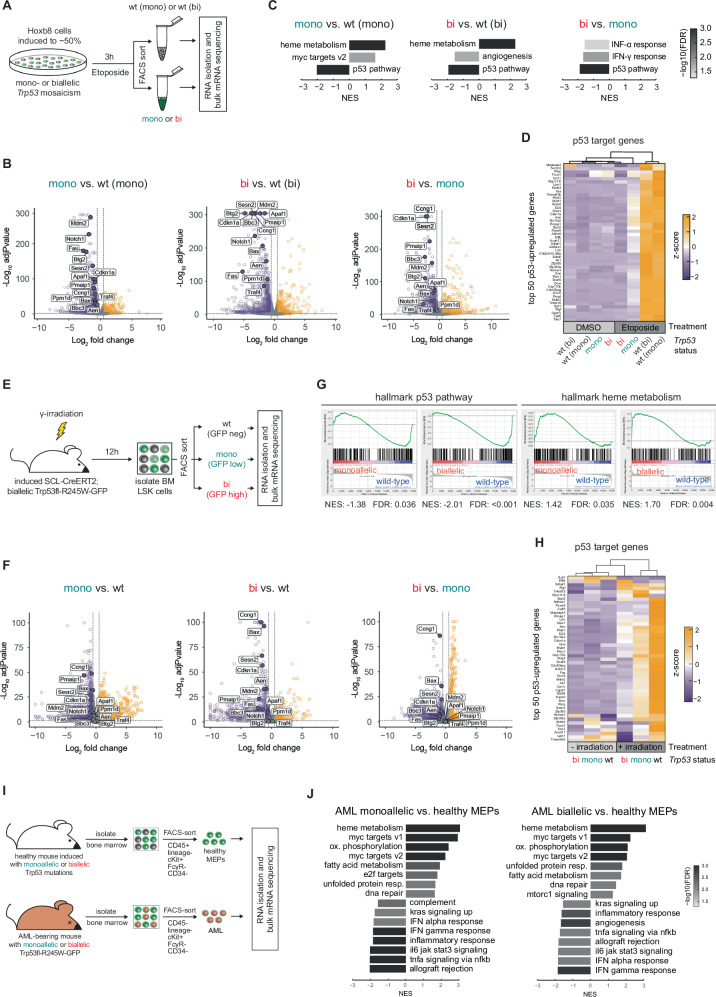
Transcriptional consequences of mono- or biallelic *Trp53* mutations in pre-malignant HSPCs and in AML blasts in vitro and in vivo. **A** Workflow for bulk mRNA sequencing of ER-HoxB8 cells. A mosaicism of ~50% *Trp53*-mutant cells was achieved by doxycycline administration, followed by treatment with DMSO or 10 µM etoposide for 3 hours. GFP^neg^ and GFP^pos^ cells were sorted, and RNA was isolated for sequencing. **B** Volcano plots of gene expression in etoposide-treated samples, highlighting 14 well-established p53 target genes (fold change cutoff = 2; *p*-value cutoff = 0.05). **C** Gene set enrichment analysis (GSEA) of the 50 hallmark gene sets in etoposide-treated samples, displaying all gene sets with significant differences (FDR < 0.05) for the indicated comparisons. **D** Heatmap of the top 50 genes most recurrently upregulated by p53 in murine RNA sequencing datasets [38], clustered hierarchically. **E** Workflow schematic for bulk mRNA sequencing of LSK cells. Following induction of *Trp53* mutations, mice were treated with γ-irradiation (250 cGy) or left untreated. 12 h later, bone marrow cells were sorted into GFP^neg^, GFP^low^, and GFP^high^ fractions within the LSK population, and RNA was isolated for sequencing. **F** Volcano plots of gene expression in γ-irradiated samples, highlighting 14 well-established p53 target genes (fold change cutoff = 2; *p*-value cutoff = 0.05). **G** Enrichment plots for the hallmark gene sets “p53 pathway” and “heme metabolism” in γ-irradiated samples. **H** Heatmap of the top 50 genes most recurrently upregulated by p53 in murine RNA sequencing datasets [38], clustered hierarchically. **I** Workflow schematic for bulk mRNA sequencing of murine AML samples. Bone marrow cells from AML-bearing mice were sorted to isolate pure AML cells, while healthy MEPs served as controls. **J** GSEA of the 50 hallmark gene sets in AML samples. The top 8 significant gene sets (FDR < 0.05) with the highest and lowest normalized enrichment scores (NES) are displayed.

Given the distinct functional consequences of mono- versus biallelic *Trp53* mutations on genomic stability and leukemic transformation, we sought to investigate the underlying mechanism, focusing on the transcriptional effects of the *Trp53* allelic state. To this end, we induced ~50% mosaicism in Hoxb8-*Trp53*^mono^ or Hoxb8-*Trp53*^bi^ cells, treated them with etoposide for 3 h, FACS-separated cells with wild-type *Trp53* from those with *Trp53*^mono^ or *Trp53*^bi^ mutations, and performed bulk mRNA sequencing (Fig. 5A). Overall, both mono- and biallelic *Trp53* mutations predominantly caused downregulation of gene expression relative to their respective wild-type controls, particularly of canonical p53 target genes such as *Cdkn1a* and *Mdm2* (Fig. 5B). Gene set enrichment analysis of the 50 hallmark gene sets revealed few significant alterations, with the “p53 pathway” showing the strongest downregulation in both *Trp53*^mono^ and *Trp53*^bi^ cells compared to their respective *Trp53*^WT^ controls (Fig. 5C). Importantly, even direct comparison between *Trp53*^bi^ and *Trp53*^mono^ cells revealed significant p53 pathway downregulation, indicating that complete p53 pathway inactivation requires biallelic *Trp53* mutations. Moreover, “heme metabolism” comprising erythroid differentiation genes, including the master regulator of erythropoiesis *Gata1*, was the most strongly upregulated gene set in cells with both mono- or biallelic *Trp53* mutations, potentially explaining the uniform erythroid phenotype of AMLs in mice (Fig. 5C). This is noteworthy as ER-Hoxb8 cells, resembling GMPs, lack erythroid potential [28]. To assess the impact of the *Trp53* allelic state on the magnitude of p53 target gene expression, we analyzed the top 50 murine p53-regulated genes from a large meta-analysis [38]. We focused our attention on genes whose expression would remain largely maintained in monoallelic *Trp53*-mutant cells but completely lost upon biallelic mutations – assuming that such genes might contribute to the observed maintenance of genomic stability and prevention of leukemic transformation in cells with monoallelic *Trp53* mutations. However, no such pattern of gene expression was discernible. Instead, expression of most p53 target genes was already strongly reduced in *Trp53*^mono^ cells (Fig. 5D).

To validate these findings in vivo, we sublethally γ-irradiated SCL-CreERT;*Trp53*-fl-R245W-GFP mice carrying biallelic *Trp53*^mutant^ alleles and sacrificed them 12 hours later. LSK cells were FACS-purified into *Trp53*^WT^, *Trp53*^mono^, and *Trp53*^bi^ populations and bulk mRNA sequencing was performed, allowing us to assess transcriptional effects of each allelic state within the same mouse (Fig. 5E). Compared to the in vitro model, we observed more differentially regulated gene sets (data not shown). However, results were concordant because “p53 pathway” and “heme metabolism” were significantly down- or upregulated, respectively, in both *Trp53*^mono^ and *Trp53*^bi^ LSK cells (Fig. 5F, G). We further confirmed the above-described pattern, with a partial loss of expression of p53 target genes in *Trp53*^mono^ cells and a near-complete loss in *Trp53*^bi^ cells (Fig. 5H).

Additionally, we examined the transcriptional changes in AML blasts versus healthy MEPs (Fig. 5I). AMLs from mono- or biallelic *Trp53* mutant mice displayed similar gene expression patterns, consistent with biallelic *Trp53* inactivation in AMLs derived from mice with monoallelic transgenes through spontaneous LOH. Genes associated with “heme metabolism” and “primitive erythroid lineage” were highly upregulated in AML blasts, further corroborating their AEL phenotype (Fig. 5J and Supplementary Fig. S8A). Importantly, the c-myc pathway known to be involved in leukemogenesis was strongly activated, while various inflammatory signatures were reduced compared to healthy MEPs (Fig. 5J and Supplementary Fig. S8B).

Collectively, these data indicate that transcriptional differences between HSPCs with mono- or biallelic *Trp53* mutations are quantitative rather than qualitative, i.e., they uniformly affect the overall magnitude of p53-regulated gene expression rather than specific gene subsets linked to genome maintenance and prevention of leukemic progression. Moreover, *Trp53* mutations appear to transcriptionally prime HSPCs for an erythroid state, eventually resulting in the development of AML with an erythroid phenotype characterized by upregulation of myc target genes.

### Non-mutational inactivation of p53 by MDM2 overexpression mimics biallelic *Trp53* mutations

**Fig. 6:**
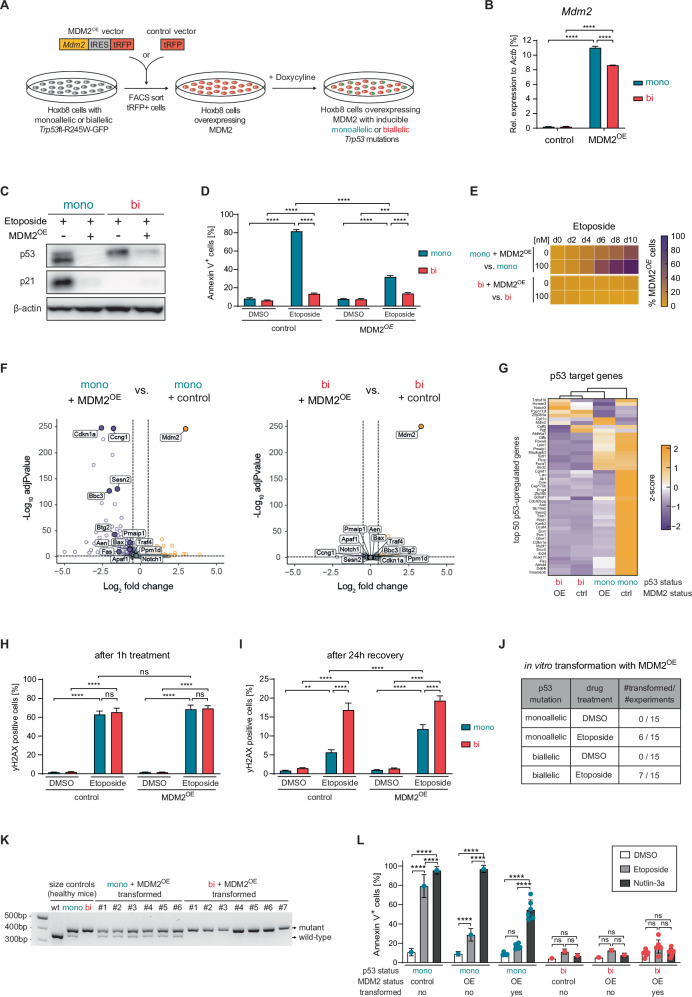
MDM2 overexpression mimics biallelic *Trp53* mutations. **A** Workflow schematic illustrating the generation of ER-HoxB8 cells overexpressing MDM2. **B** RT-qPCR analysis of *Mdm2* transcript levels in cells transduced with an MDM2 overexpression vector or a “tRFP only” control vector (biological replicates *n* = 3; symbols represent the average of experimental replicates; error bars indicate SEM; ****p < 0.0001, one-way ANOVA; Tukey multiple comparison). **C** Cells were treated with 10 µM etoposide for 3 h. Whole-cell protein lysates were resolved on a polyacrylamide gel and immunoblotted for p53, p21, and β-actin (biological replicates *n* = 3; representative image shown). **D** Cells were treated with DMSO or 1 µM etoposide for 24 h, stained with Annexin V, and analyzed by flow cytometry to determine the percentage of apoptotic cells (biological replicates *n* = 3; symbols represent the average of experimental replicates; error bars indicate SEM; ***p < 0.001, ****p < 0.0001, one-way ANOVA; Tukey multiple comparison). **E** Cells with (tRFP-positive) and without (tRFP-negative) MDM2 overexpression were mixed in a 1:9 ratio and cultured with DMSO or 100 nM etoposide for 10 days. The frequency of tRFP-positive cells was quantified every 2 days via flow cytometry (biological replicates *n* = 3). **F** Volcano plots of gene expression in etoposide-treated samples, highlighting 14 established p53 target genes (fold change cutoff = 2; *p*-value cutoff = 0.05). **G** Heatmap of the top 50 genes most frequently upregulated by p53 in murine RNA sequencing datasets [38] with hierarchical clustering. **H**, **I** Cells were treated with DMSO or 40 µM etoposide for 1 h, followed by 24 h in drug-free medium. Samples were collected immediately after the 1-h treatment and after the 24-h recovery period and γH2AX levels were assessed by flow cytometry (biological replicates *n* = 3; symbols represent the average of experimental replicates; error bars indicate SEM, **p < 0.01,****p < 0.0001, one-way ANOVA, Tukey multiple comparison). **J** Table summarizing the frequency of in vitro transformed cell line establishment in MDM2-overexpressing cells across 15 independent experiments per condition. **K** Agarose gel electrophoresis showing DNA amplicons from *Trp53*-specific PCR. **L** Cells were treated with DMSO, 1 µM etoposide or 10 µM nutlin-3a for 24 h, stained with Annexin V, and analyzed by flow cytometry to determine the percentage of apoptotic cells (biological replicates *n* = 3; symbols represent the average of experimental replicates; error bars indicate SEM; ****p < 0.0001, one-way ANOVA; Tukey multiple comparison).

Although our data revealed that biallelic *Trp53* loss-of-function (LOF) mutations are essential for leukemic transformation, clinical AML cases with only monoallelic *TP53* mutations, but sharing clinicogenomic features (e.g., complex karyotypes, poor outcomes) seen in biallelic *TP53* mutations, still occur. We hypothesized that non-mutational inactivation of the remaining *TP53* wild-type allele could contribute to leukemic transformation in such cases. To test this, we overexpressed mouse double minute 2 homolog (MDM2) – the main negative regulator of p53 [39] – in Hoxb8-*Trp53*^mono^ or Hoxb8-*Trp53*^bi^ cells to functionally model non-mutational p53 inactivation (Fig. 6A). MDM2 overexpression (MDM2^OE^) markedly increased *Mdm2* RNA and reduced p53 protein levels in both *Trp53*^mono^ and *Trp53*^bi^ Hoxb8 cells (Fig. 6B, C). In Hoxb8-*Trp53*^mono^ cells, MDM2^OE^ also reduced p21 expression and conferred resistance to apoptosis upon etoposide treatment, resulting in phenotypes resembling those of Hoxb8-*Trp53*^bi^ cells (Fig. 6C, D). Consequently, MDM2^OE^ conferred cells with monoallelic *Trp53* mutations a competitive advantage relative to *Trp53*^mono^ alone, while it had no additional clonal fitness effect in a *Trp53*^bi^ background (Fig. 6E). Bulk mRNA-seq revealed that MDM2^OE^ almost exclusively affected the transcriptome of Hoxb8-*Trp53*^mono^ cells, significantly downregulating p53 target genes to levels similar to those seen in Hoxb8-*Trp53*^bi^ cells (Fig. 6F, G).

Having established that MDM2^OE^ combined with monoallelic *Trp53* mutations mimics the *Trp53*^bi^ state in terms of basic p53-regulated functions as well as transcriptional output, we further investigated its impact on genomic stability and transformative potential. We observed increased persistence of yH2AX in Hoxb8-*Trp53*^mono^ cells with MDM2^OE^ compared to *Trp53*^mono^-only cells, though levels did not fully reach those observed in the *Trp53*^bi^ state (Fig. 6H, I). Importantly, this led nevertheless to an increased potential for in vitro transformation, as etoposide-treated cells underwent transformation not only with biallelic *Trp53* mutations, but also when MDM2^OE^ was combined with monoallelic *Trp53* mutations (Fig. 6J). Hoxb8-*Trp53*^bi^ cells with MDM2^OE^ did not transform more frequently than *Trp53*^bi^-only cells, indicating no additional transformative potential from MDM2^OE^ in cells with complete *Trp53* loss. PCR analysis revealed that these transformed *Trp53*^mono^ cell lines with MDM2^OE^ retained their wild-type *Trp53* allele (Fig. 6K). Intriguingly, this wild-type allele produced functional p53 protein as evidenced by the continued sensitivity to the MDM2 inhibitor nutlin-3a, which releases wild-type p53 from the inhibitory effect of MDM2, but resistance to etoposide, which does not affect the inhibition of wild-type p53 by MDM2 (Fig. 6L). To assess whether MDM2 overexpression is sufficient to induce a comparable p53 loss phenotype in a *Trp53* wild-type background, we repeated the analyses in *Trp53* wild-type cells and observed a similar effect (Supplementary Fig. S9A–L). These findings provide proof of concept that non-mutational p53 inactivation can recapitulate the leukemogenic consequences of biallelic *Trp53* mutations. This implies that such mechanisms could potentially be involved in the development of cases of genomically *TP53*^WT^ AML/MDS that phenotypically mimic the clinicogenomic characteristics and poor outcomes of AML/MDS with biallelic *TP53* mutations.

## Discussion

In this study, we elucidated the role of mono- or biallelic *TP53* mutations in progression from pre-malignant CH to myeloid neoplasms. Moreover, we sought to help resolve the ongoing debate surrounding the clinical and prognostic relevance of *TP53* allelic states in AML/MDS [17, 18, 20–22].

To this end, we developed novel in vitro and in vivo mouse models that allow precise control of *Trp53* mutation onset, allelic state, and clone size in HSPCs, enabling longitudinal tracking and assessment of leukemic transformation. We demonstrate a distinct relationship between the *Trp53* allelic state and basic p53 functionality in HSPCs. Monoallelic LOF mutations partially reduced p53 transcriptional activity and downstream p53-regulated biological processes, whereas biallelic *Trp53* mutations led to complete loss of p53 activity. Consequently, *Trp53*-mutant HSPCs exhibited increased clonal fitness under DNA-damaging conditions with monoallelic *Trp53* enabling expansion over wild-type cells, while still being outcompeted by biallelic mutants. Furthermore, HSPCs with monoallelic *Trp53* mutations maintained genomic integrity following DNA damage and never transformed – unless they underwent spontaneous LOH – revealing that the remaining wild-type allele retains sufficient tumor-suppressive activity to prevent leukemic transformation. In contrast, HSPCs with biallelic *Trp53* mutations accumulated genomic lesions progressed to myeloid neoplasms carrying large-scale chromosomal aberrations, recapitulating the clinical association between *TP53*-AML/MDS and complex karyotypes [17, 40].

While our findings align with the predominance of biallelic *TP53* mutations in AML/MDS, they do not fully explain the existence of clinical cases with only monoallelic mutations but clinicogenomic features such as complex karyotypes and poor prognosis typically associated with biallelic *TP53* mutations [14]. We propose three possible explanations for this phenomenon. First, accurate determination of the *TP53* allelic state in diagnostics is challenging, particularly distinguishing true monoallelic mutations from subclonal biallelic inactivation through cnLOH. Indeed, it would require WGS [41] or even single-cell analyses, which have never been conducted on a larger scale, but only in selected cases, where it led to reclassification of former monoallelic cases to the biallelic group [41, 42]. Instead, diagnostic algorithms based on the number of *TP53* mutations, their variant allele fraction and del[17p] status are currently being used in clinical routine, raising concerns about the potential for misclassification [17, 18, 20–22]. Second, non-mutational inactivation of p53 – via overexpression of negative regulators of p53 such as MDM2 or MDM4 – might explain truly monoallelic *TP53*-mutant cases that resemble the biallelic state clinicogenomically. Our findings demonstrate that MDM2 overexpression functionally mimics biallelic *Trp53* mutations, driving malignant transformation even in HSPCs retaining a wild-type *Trp53* allele. Indeed, preliminary evidence suggests that MDM2 and MDM4 overexpression occurs in AML cases with wild-type *TP53* [43, 44]. Recent data further showed that non-mutational p53 dysfunction, including MDM2 overexpression, in myeloid neoplasms retaining wild-type *TP53* leads to malignancies that share key clinical features with biallelic *TP53*-mutant cases, including equally poor survival outcomes [45]. These clinical data support our proof-of-concept findings regarding the relevance of non-mutational p53 inactivation and suggest its inclusion in diagnostic assessments to improve patient stratification. This also carries potential therapeutic implications. Patients with intact *TP53* alleles suppressed by overexpressed negative p53 regulators, such as MDM2, might benefit from therapies targeting these regulators to restore p53 function. Accordingly, we demonstrate that transformed HSPCs with monoallelic *Trp53* mutations and MDM2^OE^ retain sensitivity towards MDM2 inhibition by nutlin-3a. Third, AML/MDS cases in which *TP53* mutations are subclonal in the context of preceding recurrent AML/MDS-initiating lesions might mimic some of the clinicogenomic features of true biallelic AML/MDS evolving from *TP53*-mutant CH.

Mechanistically, our transcriptomic analyses highlight that it is a quantitative rather than qualitative difference in p53’s transcriptional activity that drive the distinct consequences of mono- or biallelic *Trp53* mutations. Monoallelic *Trp53* LOF mutations partially reduced p53 target gene expression, while loss of the remaining wild-type allele – via mutational or non-mutational mechanisms – completely abolished it. Notably, no canonical p53-regulated biological process was preferentially dysregulated on a transcriptional level in the biallelic state. This aligns with previous work in other cancer contexts, demonstrating that p53’s tumor-suppressive activity relies on its multifunctional and combined transcriptional output [46, 47].

**Fig. 7:**
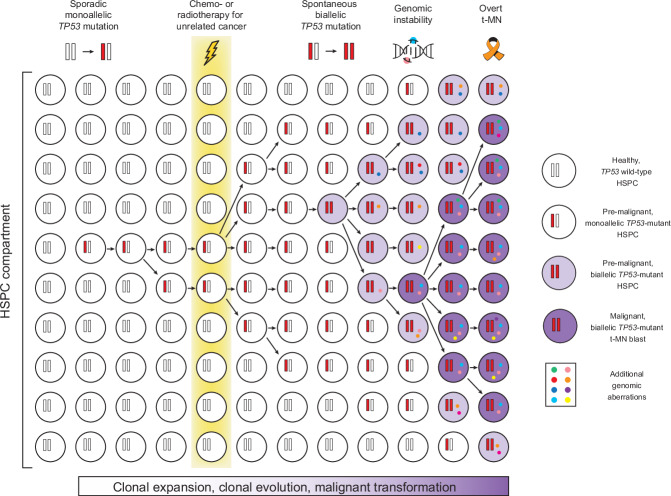
Clonal expansion, clonal evolution and malignant transformation of *TP53*-mutant CH to t-MN. Healthy HSPCs accumulate sporadic somatic mutations over time. When mutations affect *TP53*, resulting in monoallelic p53 inactivation, the affected clone gains a subtle competitive advantage over cells with wild-type *TP53*, giving rise to a pre-malignant state of CH. Exposure to chemo- or radiotherapy for an unrelated malignancy imposes a strong selective pressure, driving the rapid expansion of *TP53*-mutant HSPCs. Spontaneous loss of the remaining wild-type *TP53* allele, leading to biallelic p53 inactivation, confers further clonal fitness, accompanied by increased genomic instability. This accelerates the acquisition of additional genomic aberrations, ultimately promoting malignant transformation into leukemic blasts. As these blasts proliferate, they displace normal hematopoietic cells, disrupting blood homeostasis and resulting in overt, symptomatic t-MN.

In conclusion, to the best of our knowledge, we report the first mouse model of *TP53*-mutant therapy-related AML/MDS that recapitulates the progression from *TP53*-mutant CH to t-MN (Fig. 7), and exhibits key pathological and genomic features observed in patients. The robust AML development in this model offers a distinct advantage over existing *Trp53*-mutant mouse models, which either rarely develop AML [34, 35] or require additional oncogenic drivers not typically seen in patients with *TP53*-mutant t-AML/MDS [48–50]. Our data provide mechanistic proof for the seminal observation [12] that DNA-damaging insults like chemo- and/or radiation therapy do not directly induce *TP53* mutations but rather promote clonal expansion and leukemic progression of pre-existing HSPCs that have randomly acquired *TP53* mutations due to naturally-occurring mutagenesis during ageing (Fig. 7). Moreover, our findings support a unified classification of biallelic *TP53*-mutant AML/MDS as a distinct entity, given its association with complex karyotypes and poor prognosis. Finally, the here described well-controlled and versatile models of *TP53*-mutant t-AML/MDS will enable further mechanistic studies with the ultimate goal of preventing this devastating complication of conventional anti-cancer therapies.

## Supplementary information

Supplemental material

## Data Availability

Bulk RNA-sequencing data have been deposited in the Gene Expression Omnibus (GEO) under accession numbers GSE308508, GSE305458, GSE306212, GSE306213, and GSE299918. Whole-genome sequencing data are available in the Sequence Read Archive (SRA) under accession numbers PRJNA1309110 and PRJNA1307252.
