# Supplementary material for: The pathogenesis of therapy-related myeloid neoplasms from TP53-mutant clonal hematopoiesis
Source: Leukemia. 2025 Dec 17;40(2):279–92. doi: 10.1038/s41375-025-02839-5 (PMC12875862; doi:10.1038/s41375-025-02839-5)
Supplement: Supplementary file 1 — Supplemental material [file 41375_2025_2839_MOESM1_ESM.docx]

**SUPPLEMENTARY FIGURES**

**
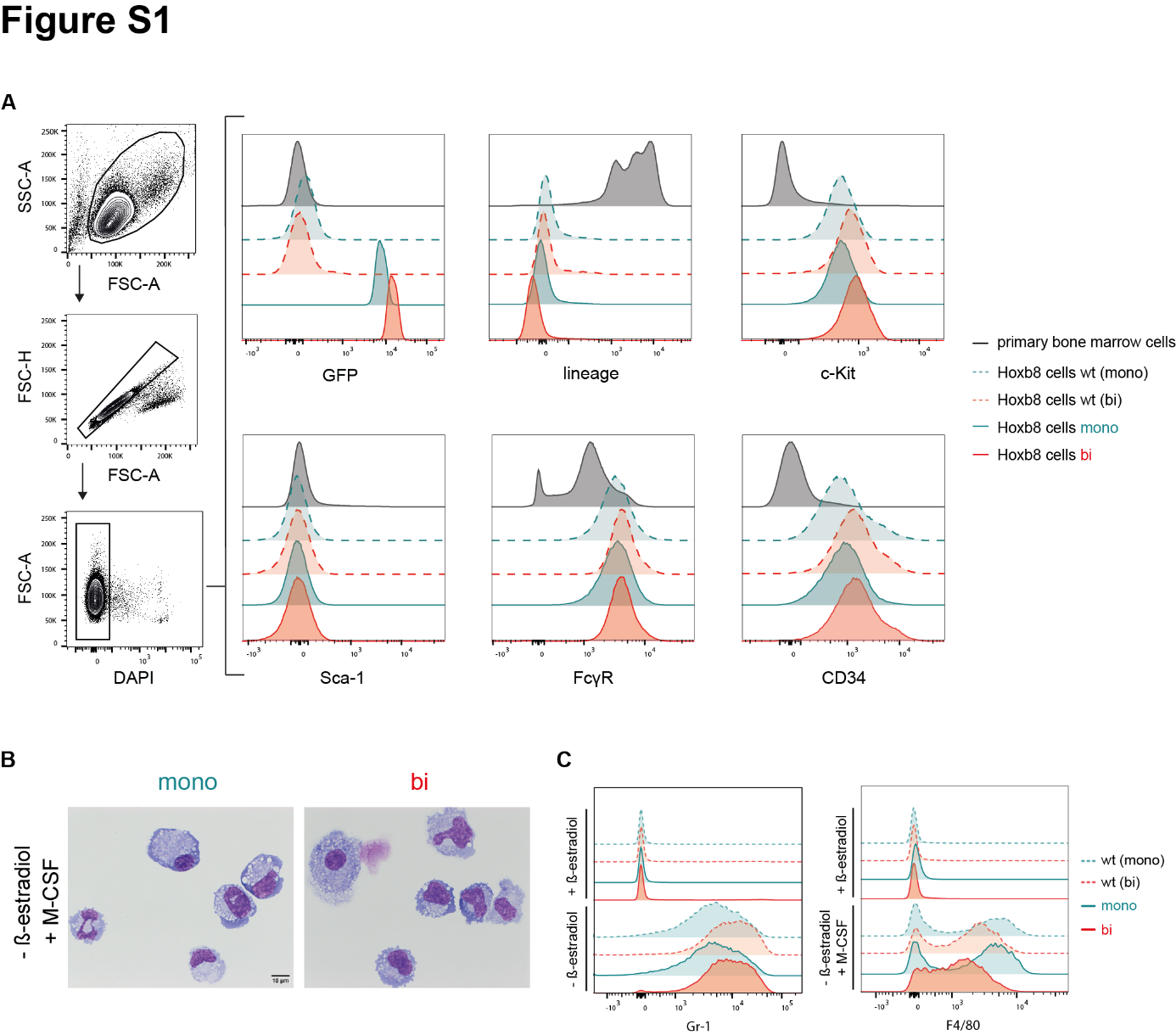
**

**Figure S1. Immunophenotype and differentiation potential of ER-HoxB8 cell lines.**

**A** ER-HoxB8 cells were stained for lineage-specific markers, and analyzed via flow cytometry. Healthy primary bone marrow cells served as controls, and representative plots are shown. **B** Cells were cultured for 6 days in media containing or lacking β-estradiol and supplemented with M-CSF. Cytospins were prepared, stained with May-Grünwald-Giemsa solution, and imaged using light microscopy; representative images are presented. **C** Flow cytometric analysis of ER-HoxB8 cells after 6 days of culture under the indicated conditions; representative flow cytometry plots are shown.

**
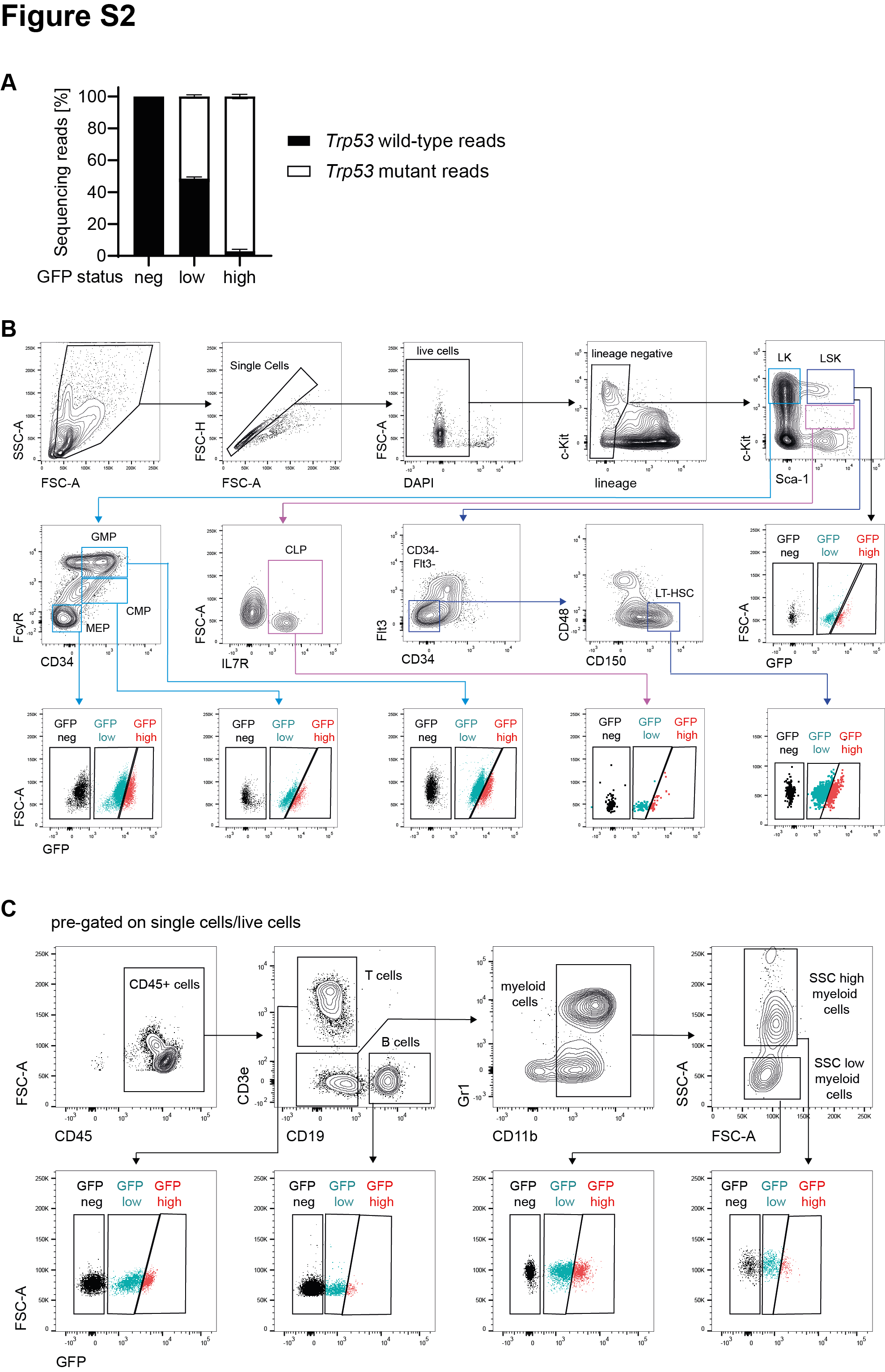
**

**Figure S2. Amplicon sequencing verification of *Trp53* recombination status and gating strategy for identifying cell populations of interest in bone marrow and peripheral blood. A** Percentage of non-recombined (*Trp53* wild-type) and recombined (*Trp53* mutant) reads determined by deep amplicon sequencing of FACS-sorted GFP^neg^, GFP^low^, and GFP^high^ LSK cells from mice harboring biallelic *Trp53*-fl-R245W-GFP alleles (n = 2; error bars represent SEM). **B** Gating strategy for identifying specific HSPC populations, as well as distinguishing GFP^neg^, GFP^low^, and GFP^high^ cell subsets. Representative plots of a healthy bone marrow sample from a biallelic *Trp53*-fl-R245W-GFP;SCL-CreERT mouse 9 weeks after injection with 5 x 100 mg/kg tamoxifen are shown. **C** Gating strategy used to identify cell populations in peripheral blood samples. Representative plots for a blood sample collected from a biallelic *Trp53*-fl-R245W-GFP;SCL-CreERT mouse 7 weeks after five 100 mg/kg tamoxifen injections are shown.

**
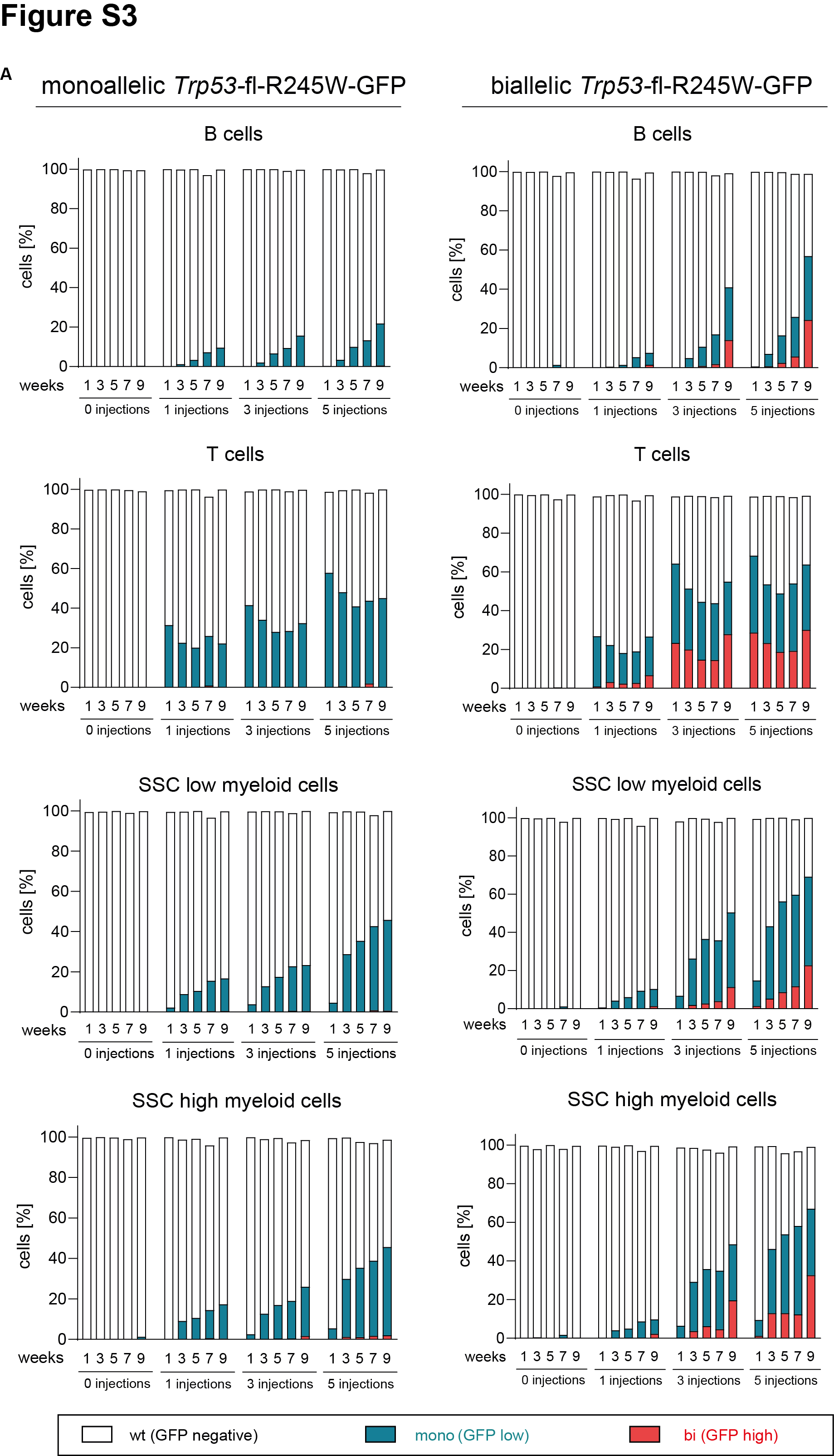
**

**Figure S3. Longitudinal analysis of GFP frequencies in blood cell populations following tamoxifen treatment. A** Mice with monoallelic or biallelic *Trp53* floxed transgenic alleles were treated with 0, 1, 3, or 5 injections of 100 mg/kg tamoxifen. Peripheral blood was collected at specified timepoints, and GFP frequencies within the indicated cell populations were analyzed by flow cytometry.


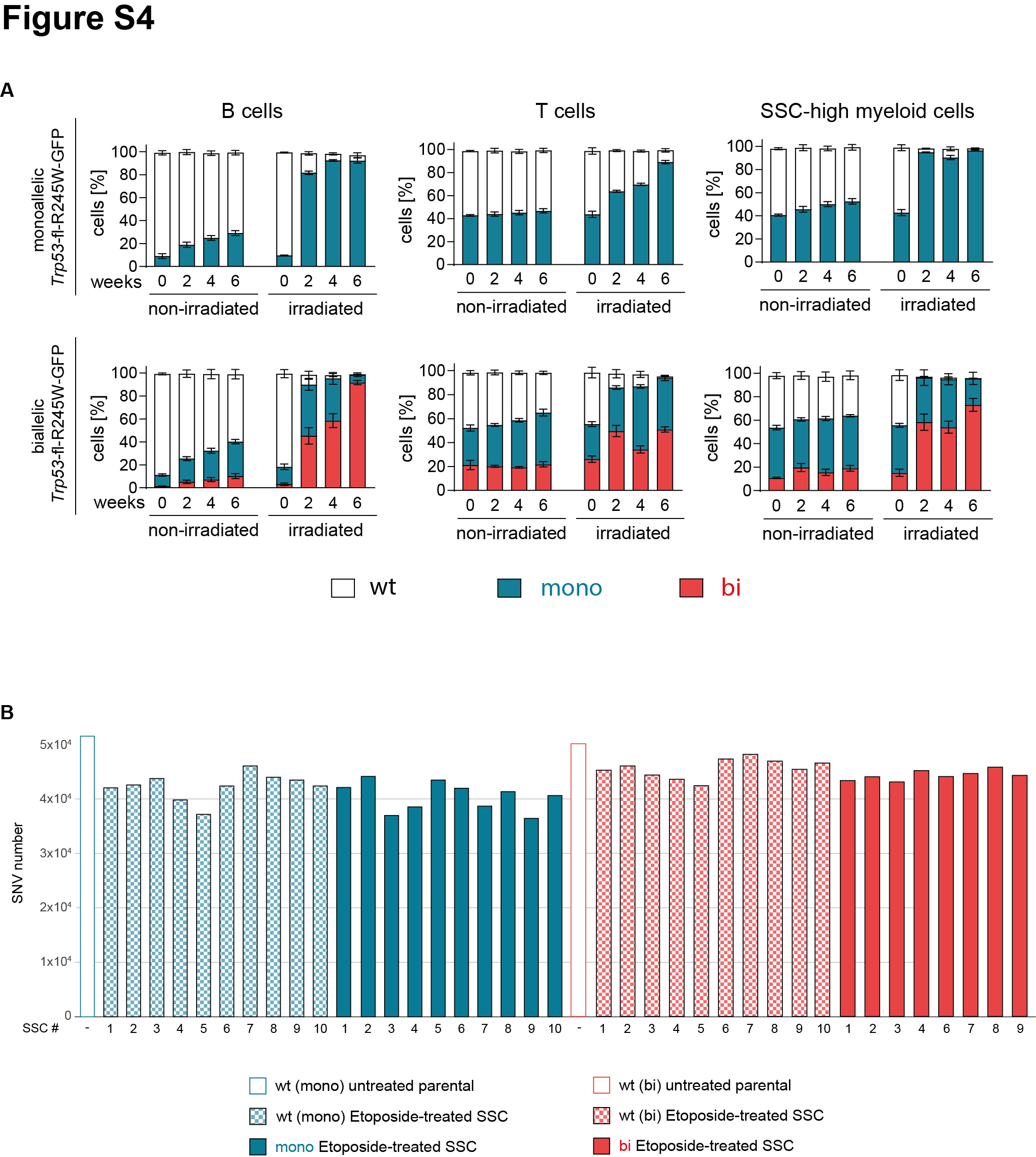


**Figure S4. *In vivo* competitive cell growth assays and SNV numbers in etoposide-treated ER-HoxB8 cells. A** Hematopoietic cells from *Trp53*-fl-R245W-GFP;SCL-CreERT mice were induced to achieve a mosaicism of 10–50% *Trp53* mutant cells across different cell subsets via tamoxifen administration. Mice were then separated into two groups: one received two sublethal doses of γ-irradiation (475 cGy) administered 4 weeks apart, while the other group was not irradiated. Peripheral blood samples were collected at the indicated time points, and GFP expression within the specified cell subtypes was analyzed by flow cytometry. **B** Single nucleotide variants (SNVs) were quantified from whole-genome sequencing of single-cell colonies derived from etoposide-treated ER-HoxB8 cells. Parental untreated bulk cells served as controls.


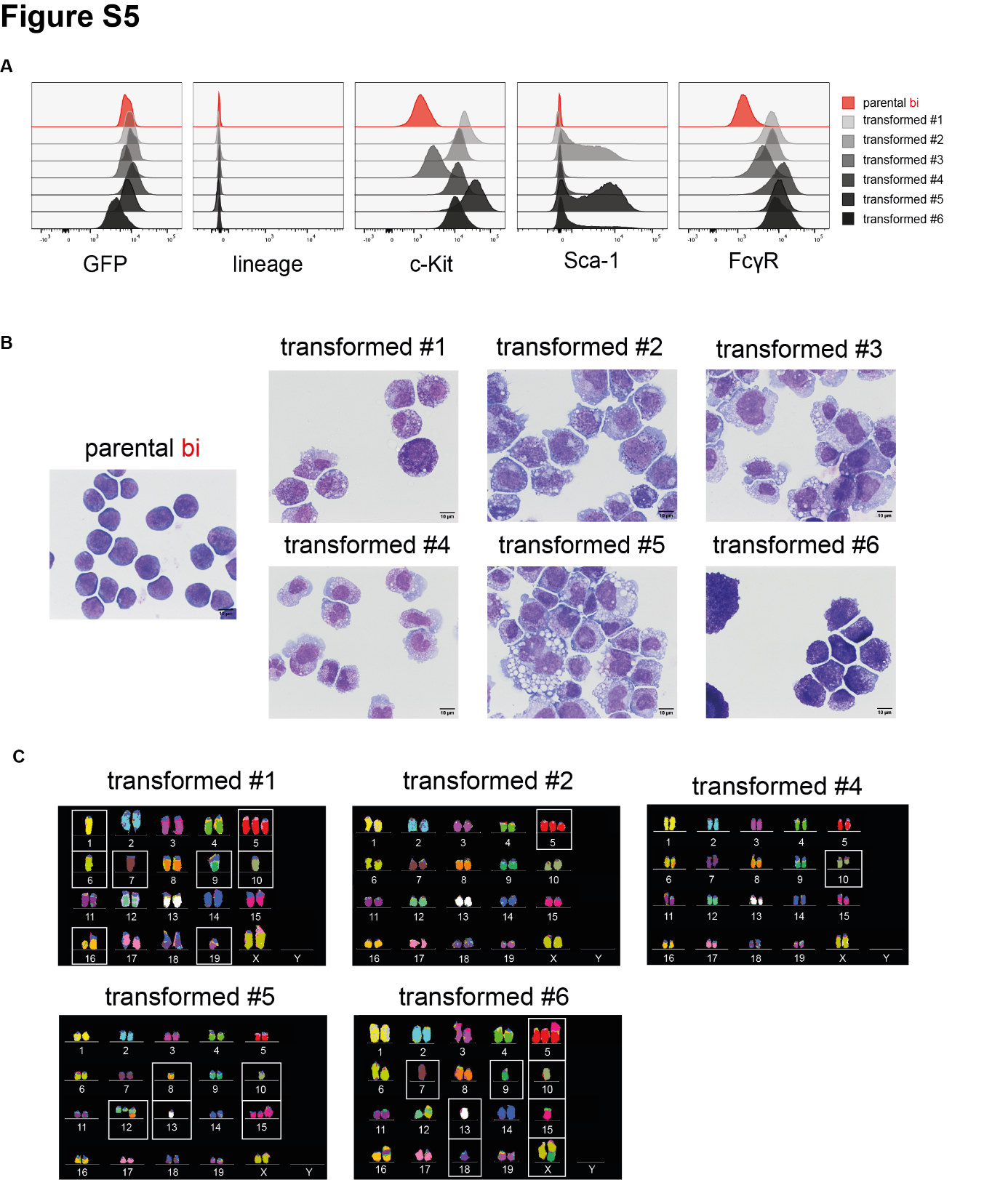


**Figure S5. Characterization of ER-HoxB8 cells after *in vitro* transformation. A** Immunophenotypic analysis of *in vitro* transformed cells by flow cytometry, compared to parental *Trp53*^bi^ ER-HoxB8 cells. **B** Cytomorphological assessment of *in vitro* transformed cells using May-Grünwald-Giemsa stained cytospins. **C** Representative metaphase spreads of *in vitro* transformed cells visualized with multicolor fluorescence in situ hybridization. Large-scale copy number alterations are highlighted.


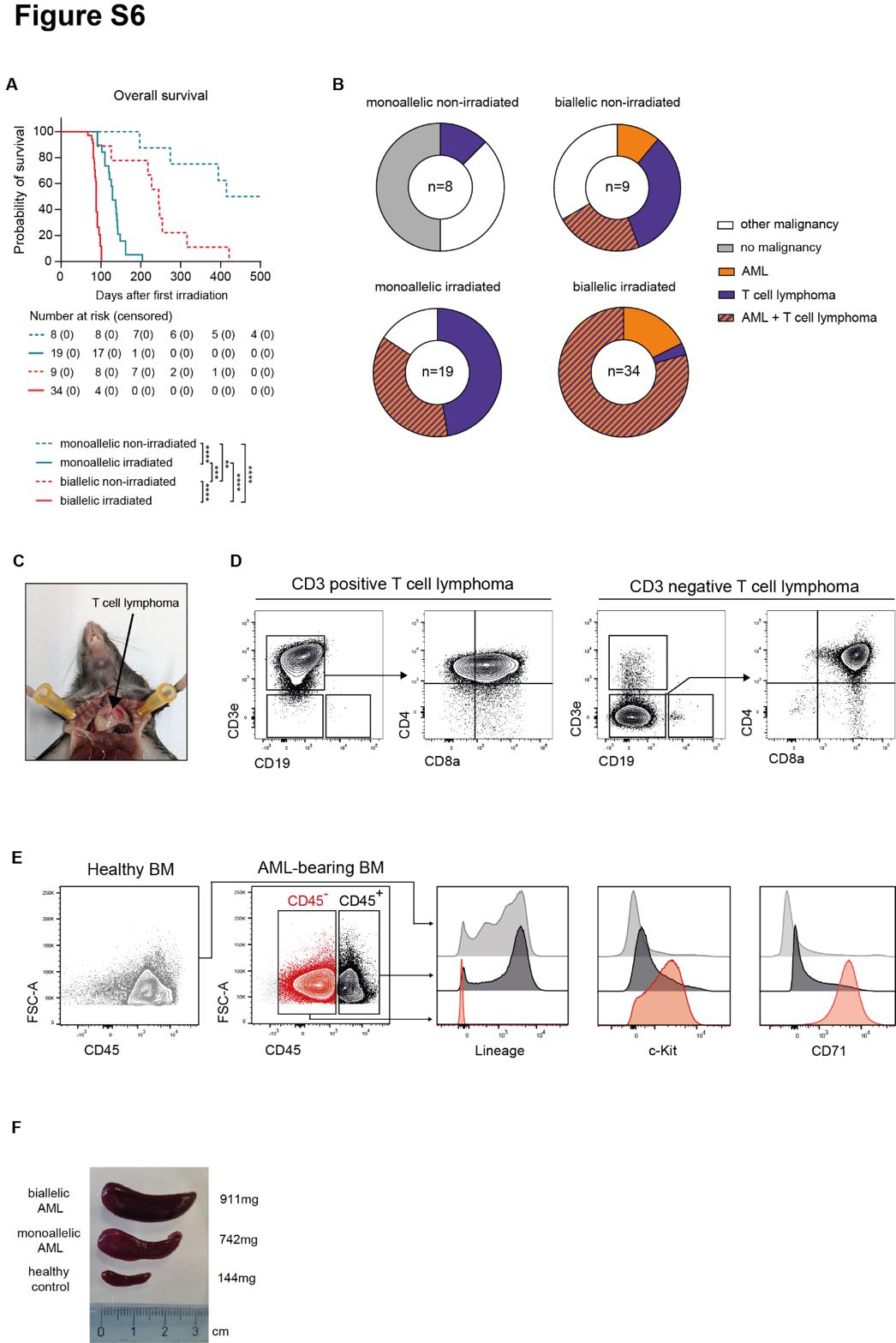


**Figure S6. Characterization of hematologic malignancies in *Trp53*-fl-R245W-GFP;SCL-CreERT mice. A** Kaplan-Meier curves showing overall survival of *Trp53*-fl-R245W-GFP;SCL-CreERT mice (**p<0.01, ***p<0.001****p<0.0001, Mantel-Cox test). **B** Spectrum of malignancies observed across the indicated experimental groups. Dashed pattern indicates the presence of both malignancies occurring simultaneously in the same mouse. **C** Photograph illustrating the localization of thymic T cell lymphomas. **D** Two representative immunophenotypes of T cell lymphomas, showing variable CD3 expression and robust double-positivity for CD4 and CD8a. **E** Representative immunophenotype of acute erythroid leukemia, characterized by CD45 negativity and high expression of c-Kit and CD71. CD45 gating was applied to distinguish blasts from residual normal BM cells. BM from a mouse without malignancy is included for comparison. **F** Photograph showing representative spleen sizes of AML-bearing and healthy control mice.


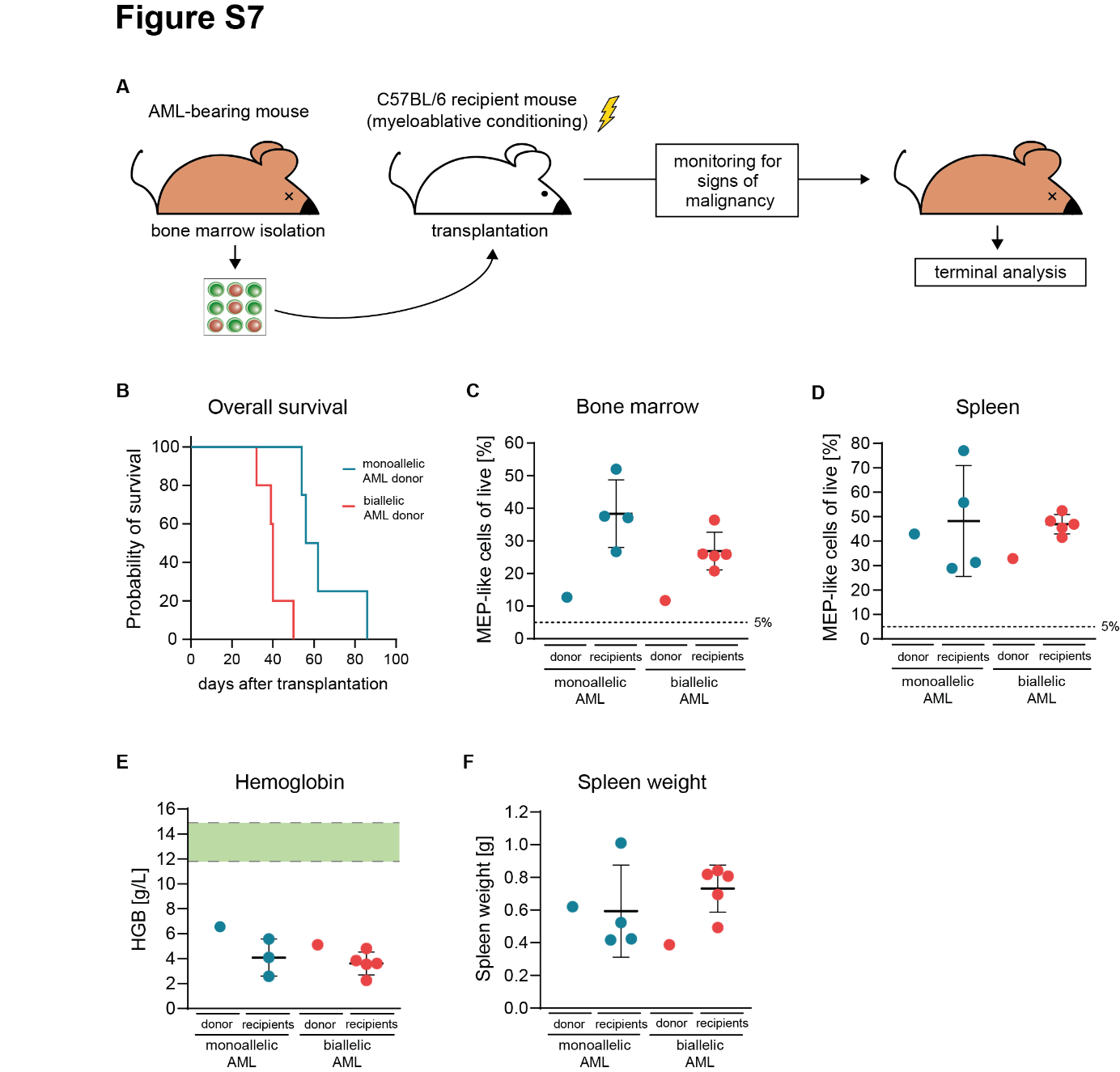


**Figure S7. Disease-initiating potential of transplanted AML cells derived from *Trp53*-fl-R245W-GFP;SCL-CreERT mice. A** Schematic of the experimental workflow for bone marrow transplantation experiments. Whole bone marrow cells from AML-bearing mice were transplanted into myeloablative-conditioned C57BL/6 mice. Mice were terminated upon the onset of malignancy-related symptoms. **B** Survival curves of recipient mice following bone marrow transplantation. **C** Frequency of MEP-like cells in bone marrow of recipient mice, compared to the corresponding AML-bearing donor mice. **D** Frequency of MEP-like cells in spleens of donor and recipient mice. **E** Blood hemoglobin values at the termination point in both donor and recipient mice, with the normal range indicated by the green bar. **F** Spleen weight of donor and recipient mice at the time of termination.


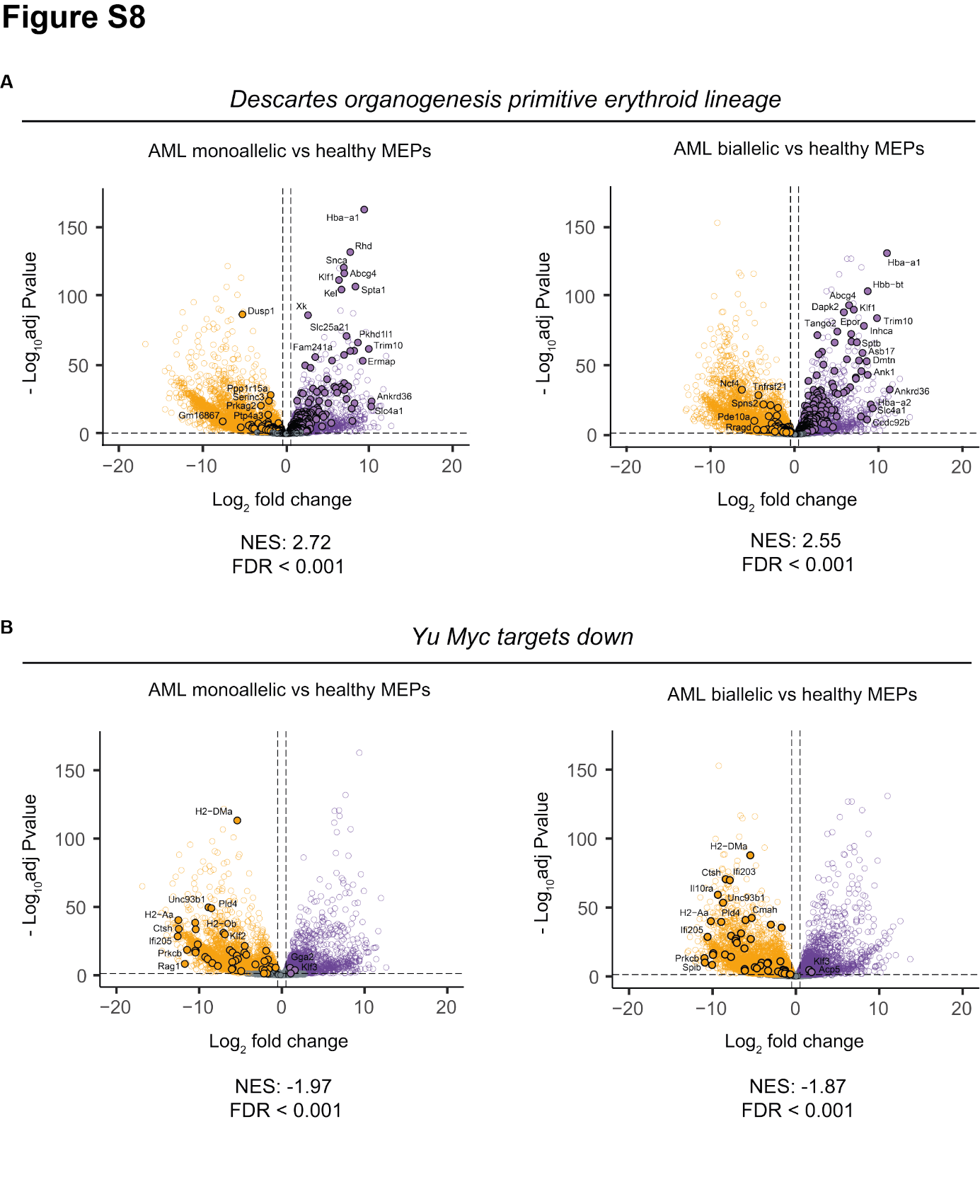


**Figure S8. Transcriptomic analysis of murine AMLs. A** Volcano plots of gene expression in AML samples versus healthy MEPs. Genes in the “Descartes organogenesis primitive erythroid lineage” gene set are highlighted (fold change cutoff = 2; p-value cutoff = 0.05) and NES and FDR values of corresponding gene set enrichment analyses are shown. **B** Volcano plots with genes in the “Yu Myc targets dn” gene set highlighted (fold change cutoff = 2; p-value cutoff = 0.05). NES and FDR values of corresponding gene set enrichment analyses are shown.

**
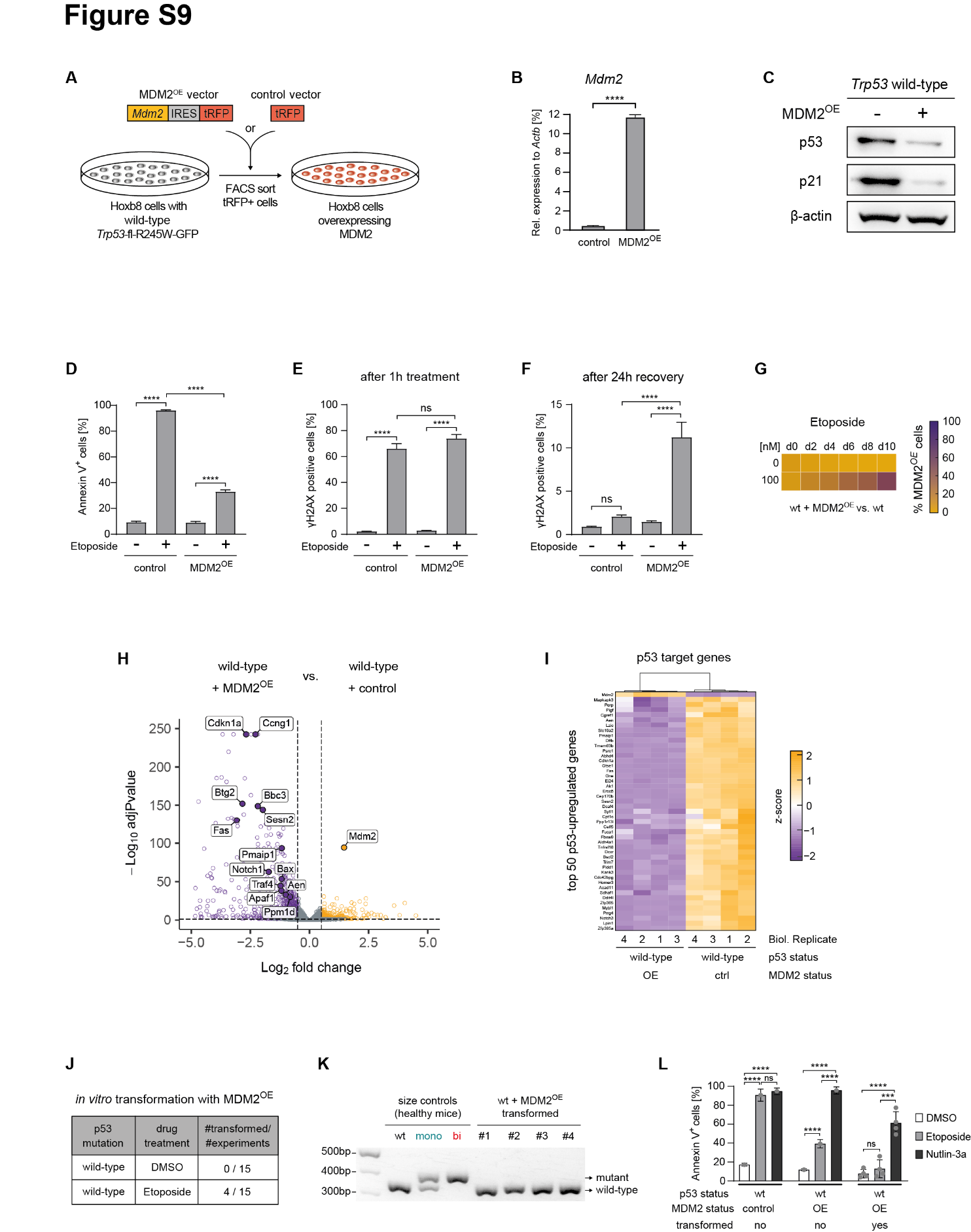
**

**Figure S9. MDM2 overexpression in *Trp53* wild-type cells mimics biallelic *Trp53* mutations. A** Workflow schematic illustrating the generation of *Trp53* wild-type ER-HoxB8 cells overexpressing MDM2. **B** RT-qPCR analysis of *Mdm2* transcript levels in cells transduced with an MDM2 overexpression vector or a “tRFP only” control vector (biological replicates n = 3; symbols represent the average of experimental replicates; error bars indicate SEM; ****p < 0.0001, one-way ANOVA; Tukey multiple comparison). **C** Cells were treated with 10 µM etoposide for 3 hours. Whole-cell protein lysates were resolved on a polyacrylamide gel and immunoblotted for p53, p21, and β-actin (biological replicates n = 3; representative image shown). **D** Cells were treated with DMSO or 1 µM etoposide for 24 hours, stained with Annexin V, and analyzed by flow cytometry to determine the percentage of apoptotic cells (biological replicates n = 3; symbols represent the average of experimental replicates; error bars indicate SEM; ****p < 0.0001, one-way ANOVA; Tukey multiple comparison). **E-F** Cells were treated with DMSO or 40 µM etoposide for 1 hour, followed by 24 hours in drug-free medium. Samples were collected immediately after the 1-hour treatment and after the 24-hour recovery period and γH2AX levels were assessed by flow cytometry (biological replicates n=3; symbols represent the average of experimental replicates; error bars indicate SEM, **p<0.01,****p<0.0001, one-way ANOVA, Tukey multiple comparison). **G** Cells with (tRFP-positive) and without (tRFP-negative) MDM2 overexpression were mixed in a 1:9 ratio and cultured with DMSO or 100 nM etoposide for 10 days. The frequency of tRFP-positive cells was quantified every 2 days via flow cytometry (biological replicates n = 3). **H** Volcano plot of gene expression in etoposide-treated samples, highlighting 14 established p53 target genes (fold change cutoff = 2; p-value cutoff = 0.05). **I** Heatmap of the top 50 genes most frequently upregulated by p53 in murine RNA sequencing datasets (1) with hierarchical clustering. 4 biological replicates per group shown. **J** Table summarizing the frequency of *in vitro* transformed cell line establishment in MDM2-overexpressing cells across 15 independent experiments per condition. **K** Agarose gel electrophoresis showing DNA amplicons from *Trp53*-specific PCR. **L** Cells were treated with DMSO, 1 µM etoposide or 10 µM nutlin-3a for 24 hours, stained with Annexin V, and analyzed by flow cytometry to determine the percentage of apoptotic cells (biological replicates n = 3; symbols represent the average of experimental replicates; error bars indicate SD; ***p < 0.001, ****p < 0.0001, one-way ANOVA; Tukey multiple comparison).

**SUPPLEMENTARY METHODS**

**Nomenclature for *Trp53* status used in the manuscript**

To minimize confusion and ensure consistency across model systems, we have adopted a simplified nomenclature that always refers to the a priori experimentally-induced *Trp53* status:

**wt**: cells carrying two functionally *Trp53* wild-type alleles, i.e., transgenic alleles prior to Cre-mediated recombination of the *Trp53-fl*-R245W-GFP locus. wt (mono) or wt (bi) indicate whether the cells carry one (*Trp53*-fl-R245W-GFP/+) or two (*Trp53*-fl-R245W-GFP / *Trp53*-fl-R245W-GFP) non-recombined transgenic alleles, respectively.

**mono**: cells carrying one experimentally-induced Trp53-R245W mutation and one functional wild-type allele. The functional wild-type allele can either be a normal wild-type allele (as in Hoxb8 cells or mice with *Trp53*-fl-R245W-GFP/+) or a non-recombined transgenic allele (corresponding to the GFP^low^ population in mice with *Trp53*-fl-R245W-GFP / *Trp53*-fl-R245W-GFP).

**bi**: cells carrying two experimentally-induced biallelic *Trp53*-R245W mutations (*Trp53*-fl-R245W-GFP / *Trp53*-fl-R245W-GFP), resulting in complete loss of functional wild-type p53.

**Generation and handling of ER-HoxB8-tetCre-*Trp53*-fl-R245W-GFP cell lines**

Conditionally immortalized murine Hoxb8 cell lines were generated as previously described (2). Whole bone marrow cells were isolated from 14-week-old female littermates carrying the *Trp53*-fl-R245W-GFP/+ and *Trp53*-fl-R245W-GFP/*Trp53*-fl-R245W-GFP transgenes. Cells were cultured in pre-stimulation medium (RPMI supplemented with 10% fetal bovine serum (FBS), 1% penicillin-streptomycin, 4% SCF-conditioned medium, 10 ng/mL recombinant murine IL-3 Protein (Peprotech, Cat. No. 213-13), and 10 ng/mL recombinant murine IL-6 Protein (Cat. No. 406-ML) for 48 hours. Subsequently, cells were transduced with a retrovirus containing the MSCVneo-HA-ER-Hoxb8 plasmid (a gift from Mark Kamps & David Sykes; RRID:Addgene_222291). Transduced cells were selected with 1 mg/mL geneticin (G-418, Sigma Aldrich, Cat. No. 4727878001) for one week.

In a next step, the TRE-Cre-PuroR-rtTA plasmid, containing a doxycycline-inducible Cre recombinase (derived by cloning Cre recombinase from pENTR-Cre (a gift from Minoru Ko; RRID:Addgene_139545), into pCW57.1 (a gift from David Root; RRID:Addgene_41393)), was introduced via lentiviral transduction. Transduced cells were selected with 2 mg/mL puromycin (InvivoGen, Cat. No. ant-pr-1) for two weeks. To eliminate the small fraction of cells undergoing Cre-mediated *Trp53*-fl-R245W-GFP recombination in the absence of doxycycline, GFP negative cells were sorted using a FACS Aria III 4L (Becton Dickinson, Franklin Lakes, NJ, USA).

For experimental induction of the *Trp53*-R245W mutation, cells were treated with up to 2.5 μg/mL Doxycycline-hyclat (Sigma Aldrich, Cat. No. D9891) for 48 hours. When pure populations of wild-type or mutant cells were required, cells were sorted by FACS into GFP-negative and GFP-positive fractions, respectively.

Cells were cultured in RPMI supplemented with 10% FBS, 1% penicillin-streptomycin, 2% SCF-conditioned medium (produced by Chinese hamster ovary cells stably secreting SCF), and 0.5 μM β-estradiol (Sigma Aldrich, Cat. No. E2758). All cultures were maintained at 37°C in a humidified atmosphere of 95% air and 5% CO₂ and were passaged every 2–3 days. Early-passage cells (P3–P4) were cryopreserved and fresh cells were thawed every 6–8 weeks to ensure low passage numbers during experiments. Cultures were tested for mycoplasma contamination and confirmed negative.

**Retroviral transduction**

HEK293T cells (RRID:CVCL_0063) were transfected with transfer plasmids encoding the DNA sequence of interest along with the pCL-eco retroviral packaging vector (a gift from Inder Verma; RRID:Addgene_12371). Transfections were performed using JetPRIME® transfection reagent (Polyplus Transfection, Illkirch, France) to enhance efficiency. The medium was replaced 24 hours post-transfection, and viral particles were collected 24 hours later.

For transduction, 1 x 10^6^ target cells were exposed to 1–2 mL of freshly harvested viral particles in 12-well plates pre-coated with retronectin (Takarabio, Cat. No. T100). Transductions were conducted in the presence of 8 μg/mL polybrene (Sigma Aldrich®, Cat. No. TR-1003-G) using spinoculation at 1,000 x g and 32°C for 90 minutes.

**Lentiviral transduction**

Replication-defective, third-generation lentiviral vectors were generated by transfecting HEK293T cells with the transfer plasmid encoding the DNA sequence of interest, along with the psPAX2 (RRID:Addgene_12260) packaging plasmid and the pCAG-VSVG (RRID:Addgene_64084) envelope plasmid (both kindly provided by Dr. Patrick Salmon, University of Geneva, Switzerland). Transfections were performed using JetPRIME® transfection reagent (Polyplus Transfection, Illkirch, France) to enhance efficiency. The culture medium was replaced 24 hours after transfection, and viral particles were harvested 24 hours later.

For transduction, 1 x 10^6^ target cells were incubated with 1–2 mL of freshly collected viral particles in the presence of 8 μg/mL polybrene (Sigma Aldrich®, Cat. No. TR-1003-G). Transductions were carried out via spinoculation at 1,000 × g and 32°C for 90 minutes.

**Overexpression of MDM2**

A lentiviral SFFV-*Mdm2*-IRES-tRFP transfer plasmid was constructed by PCR amplification of the Mdm2-IRES sequence derived from MSCV-Mouse *Mdm2* wt-IRES-CFP (a gift from Martine Roussel; RRID:Addgene_180493) with ClaI and NcoI restriction site overhangs. The amplified fragment was cloned into the sgRNA.SFFV.tRFP backbone (a gift from Benjamin Ebert; RRID:Addgene_169941). ER-HoxB8-tetCre-*Trp53*-fl-R245W-GFP cells were transduced with the resulting lentiviral plasmid. Successfully transduced cells were enriched by FACS based on tRFP expression.

**Flow cytometry**

For surface staining, the FACS antibodies listed in table 1 were used at the indicated dilutions and incubated for 30-60 minutes at 4°C. DAPI (4’,6-diamidino-2-phenylindole dilactate; BioLegend, Cat. No. 422801) or Fixable Viability Dye (FVD) eFluor™ 780 (eBioscience™, San Diego, CA, USA) were used for live/dead cell discrimination. Data acquisition was carried out using an LSRFortessa™ 4L flow cytometer equipped with a high-throughput sampler (HTS) (Becton Dickinson, Franklin Lakes, NJ, USA). Analysis and quantification of data were performed using FlowJo® v9x software (RRID:SCR_008520, Becton Dickinson, Franklin Lakes, NJ, USA). FACS was conducted on an Aria III 4L (Becton Dickinson, Franklin Lakes, NJ, USA).

**Differentiation assay**

A total of 3 x 10⁶ ER-HoxB8-tetCre-*Trp53*-fl-R245W-GFP cells with the indicated genotypes were washed twice with PBS and resuspended in one of three conditions: (I) normal culture media (+β-estradiol, + SCF), (II) culture media without β-estradiol, but with SCF, or (III) culture media without β-estradiol, without SCF, but supplemented with 10 ng/mL murine M-CSF (Biolegend, Cat. No. 576402). Cells were then plated in non-tissue culture-treated 10 cm dishes and cultured for 6 days. Cells in the normal culture media condition were split 1:8 every two days to maintain optimal cell density. After 6 days, non-adherent cells were collected by pipetting, and adherent cells were harvested via trypsinization. Cells were subsequently stained for lineage-defining surface markers and analyzed by flow cytometry, as well as subjected to cytomorphological analysis.

**Chemical compounds**

Etoposide (Selleckchem, S1225), nutlin-3a (Selleckchem, S8059), daunorubicin (Selleckchem, S3035), and cytarabine (Selleckchem, S1648) were procured as lyophilized formulation, dissolved in Dimethyl Sulfoxide (DMSO, Sigma-Aldrich®, D4540), aliquoted, and stored at -80°C. For all experiments, the drugs were freshly thawed and diluted in cell culture medium to achieve the required concentrations.

**Cell cycle analysis**

Asynchronous cultures of ER-HoxB8-tetCre-*Trp53*-fl-R245W-GFP cells were seeded in 6-well plates at a density of 1.5 x 10^6^ cells per well and treated with 40 μM etoposide for 1 hour. Following treatment, the drug was removed by washing the cells twice and cells were cultured in fresh media for 24 hours. A total of 1.5 x 10^5^ viable cells were collected, transferred to 96-well plates, and stained with 1:200 CytoPhase™ Violet (BioLegend, Cat. No. 425701) in culture media for 90 minutes at 37°C. Cell cycle distribution was assessed using flow cytometry on a BD LSRFortessa™ system (BD Biosciences). Data analysis was performed with FlowJo software (v10.0.7, FlowJo, LLC, Ashland, Oregon, USA).

**Apoptosis assay**

ER-HoxB8-tetCre-*Trp53*-fl-R245W-GFP cells were seeded at a density of 1.5 x 10^5^ cells per well in 12-well plates and treated with 10 μM etoposide or vehicle. After 24 and 48 hours, 200μl of cell suspension were removed and transferred to a 96-well plate for analysis. Cells were stained in Annexin V Binding Buffer (Biolegend, Cat. No. 422201) with 1:20 APC Annexin V (Biolegend, Cat. No. 640920) in combination with 1:2500 DAPI (4’,6-15 Diamidino-2-Phenylindole Dilactate; Invitrogen, Cat. No. D1306) or 1:1000 Fixable Viability Dye eFluor780 (eBioscience, Cat. No. 65-0865-14) for 15min at RT. Data was acquired by flow cytometry (BD LSRFortessa) and analyzed with FlowJo software (v10.0.7, FlowJo, LLC, Ashland, OR, USA)

**Immunoblots**

Whole-cell extracts were prepared using Pierce™ RIPA lysis buffer (Thermo Scientific, Cat. No. 89900), freshly supplemented with cOmplete™ protease inhibitor cocktail (Sigma Aldrich, Cat. No. 5892791001). Protein concentrations were determined using the Pierce™ BCA Protein Assay Kit (Thermo Scientific, Cat. No. 23225), with absorbance measured at 562 nm. Equal amounts of protein were loaded onto either NuPAGE™ 4–12% Bis-Tris Midi gels (Invitrogen, Cat. No. WG1403BOX, 1.0 mm) or Bolt™ 4–12% Bis-Tris Plus Mini gels (Invitrogen, Cat. No. NW04125BOX, 1.0 mm, WedgeWell™ format). Proteins were transferred to Amersham™ Hybond® P PVDF membranes (Merck, Cat. No. GE10600023) and subjected to immunoblotting with primary antibodies, followed by HRP-conjugated secondary antibodies. The antibodies used were anti-p21 (EPR18021, RRID:AB_2734729, dilution 1:1000), anti-p53 (1C12, RRID:AB_331743, dilution 1:1000), anti-β-actin (Abcam, RRID:AB_306371, dilution 1:5000), goat anti-mouse-HRP (Genesee Scientific, Cat. No. 20-304, dilution 1:5000) and goat anti-rabbit-HRP (Genesee Scientific, Cat. No. 20-303, dilution 1:5000). Blots were visualized using Immobilon Forte HRP substrate (Merck, Cat. No. WBLUF0100) and imaged using the Fusion Solo S Western Blot imager (Vilber).

**Quantitative reverse transcription PCR**

Total RNA was extracted using the RNeasy Kit (QIAGEN, Cat. No. 74104) following the manufacturer’s protocol. Reverse transcription was performed with High-Capacity cDNA Reverse Transcription Kit (Applied Biosystems, Cat. No. 4368814). qPCR was conducted using the TaqMan® Gene Expression Master Mix (ThermoFisher, Cat. No. 4369016) and TaqMan® Gene Expression Assays (Cdkn1a, Mm00432448_m1; Mdm2, Mm01233136_m1, Actb, Mm00607939_s1) on a ViiA 7 Real-Time PCR System (Applied Biosystems) or 7500 Fast Real-Time PCR System (Applied Biosystems). The relative mRNA expression of each target gene was normalized to the expression of β-actin.

**Drug sensitivity assays**

For dose-response curve generation, cells were seeded in opaque 96-well flat-bottom culture plates at a density of 0.8 x 10⁴ cells per well. Serial concentrations of etoposide, nutlin-3a, daunorubicin, and cytarabine were dispensed using an HP® D300e Digital Dispenser (HP Inc., Tecan Life Sciences). Plates were sealed with breathable foil (ThermoFisher Scientific, Nunc sealing tape, white Rayon, breathable, sterile, Cat. No. 241205) to minimize evaporation. Following 48 hours of drug exposure, cell viability was measured using the CellTiter-Glo luminescent assay (Promega, Cat. No. G7572) and luminescence was recorded with a Biotek Synergy LX (SLXFTS) luminometer (Agilent technologies). Dose-response curves were generated by normalizing cell viability to vehicle-treated controls and fitting the data using nonlinear regression analysis. For statistical analysis, the areas under the dose-response curves between cells with different genotypes were compared using GraphPad Prism software (RRID:SCR_002798, version 10.2.1, Boston, MA, USA).

**Competitive cell growth assay**

ER-HoxB8-tetCre-*Trp53*-fl-R245W-GFP cells were seeded at a density of 1.5 x 10⁴ cells per well in flat-bottom 96-well plates with a genotype chimerism of 10%. To compare the effects of different *Trp53* allelic states, cells were pre-treated with doxycycline (80–320 ng/mL) for 72 hours to establish a 10% chimerism. For experiments evaluating the impact of MDM2 overexpression, induced cells were sorted by FACS to isolate pure tRFP/GFP-positive populations, which were then mixed at a 1:9 ratio. Drugs or DMSO were added to a final volume of 200 μL per well. Every 48 hours, 100 μL of the cell suspension was transferred to a V-bottom 96-well plate for analysis on a BD LSRFortessa™ Flow Cytometer with a High Throughput Sampler (HTS; Becton Dickinson, Franklin Lakes, NJ, USA). Wells with high cell density were diluted up to 1:4, and fresh culture medium containing the appropriate drug concentrations was replenished to maintain consistent exposure.

**γH2AX staining**

For flow cytometric γH2AX analysis, ER-HoxB8-tetCre-*Trp53*-fl-R245W-GFP cells were pre-treated with doxycycline (1,000–2,500 ng/mL) for 72 hours to achieve a genotype chimerism of 30–50%. Cells were then seeded at a density of 1 x 10⁶ cells per well in 12-well plates and exposed to etoposide (10–40 μM) or DMSO for 1 hour at 37°C. After treatment, 150 μL of the cell suspension was used for staining and flow cytometric analysis. The remaining cells were washed twice with PBS and incubated for 24 hours in fresh, drug-free culture medium in 12-well plates, after which up to 1.5 x 10⁵ live cells were collected for γH2AX staining and analysis. For intracellular staining, cells were fixed and permeabilized with Cytofix/Cytoperm Fixation/Permeabilization Kit (BD Biosciences, Cat. No. 554714) and incubated with a biotin conjugated anti-Phospho-Histone H2A.X (ser139) antibody (Clone JBW301, RRID:AB_310795, 1:250 dilution) for 45 minutes, followed by staining with Streptavidin-APC (Biolegend, Cat. No. 405207, 1:133 dilution) for 30 minutes. Cells were then resuspended in FACS buffer containing DAPI (BioLegend, Cat. No. 422801,1:2500 dilution) and analyzed using a BD LSRFortessa™ Flow Cytometer equipped with a High Throughput Sampler (HTS; Becton Dickinson, Franklin Lakes, NJ, USA).

**In *vitro* transformation assay**

A total of 1.5 x 10⁶ ER-HoxB8-tetCre-*Trp53*-fl-R245W-GFP cells of the specified genotypes were treated with 40 μM etoposide or DMSO for 1 hour. Following treatment, cells were washed twice with PBS and cultured in fresh, drug-free culture medium until the recovery of at least 1.5 x 10⁶ viable cells was achieved, typically within 4–5 days. This treatment cycle was then repeated twice more for a total of three chemotherapy cycles. After completing the chemotherapy regimen, β-estradiol was progressively removed from the culture medium by reducing its concentration by half every two days. After a total of 12 days, cells were cultured in β-estradiol-free medium. Subsequently, cells were monitored for their ability to grow independently of β-estradiol.

**Cytomorphology**

For cytological analysis, 100 μL of cell suspension (2 x 10⁶ cells/mL) were applied onto Cytofunnel slides via centrifugation at 600 × g for 5 minutes. The cells were subsequently stained with May-Grünwald-Giemsa solution according to the manufacturer’s protocol, followed by examination and imaging using light microscopy on a Zeiss Axio Imager A2.

**Histology**

Murine femora were fixed in 4% paraformaldehyde and subsequently embedded in paraffin. Tissue sections were prepared at a thickness of 2 µm using a rotatory microtome (Microm HM355S, Thermo Fisher Scientific) and hematoxylin/eosin staining was performed at the Department of Pathology and Molecular Pathology, University Hospital of Zurich. Images of the stained slides were captured using a Zeiss Axio Imager A2 microscope.

**Karyotyping**

Metaphase preparation was performed following standard protocols. Multicolor fluorescence in situ hybridization (mFISH) was carried out according to the manufacturer’s instructions for the 21XMouse mFISH probe (MetaSystems GmbH, Altlussheim, Germany; Cat. No. D-0425-060-DI), which targets all chromosomes. Briefly, metaphases were dropped onto slides and denatured in a salt solution. Separately, 6 µL of the mFISH probe were denatured and applied to the metaphase spreads. The hybridization area was covered with an 18 x 18 mm coverslip and sealed with Fixogum Rubber Cement (Lucerna Chem AG, Luzern, Switzerland). Hybridization was conducted overnight at 37°C under humid conditions using a HYBrite system (Abbott Molecular/Vysis, Baar, Switzerland). Non-hybridized probe was removed by washing the slides in 0.4x saline-sodium citrate (SSC) buffer with 0.3% (vol/vol) IGEPAL (Sigma Aldrich) for 120 seconds at 72°C ± 2, followed by a wash in 2x SSC buffer with 0.1% (vol/vol) IGEPAL for 60 seconds at room temperature. Slides were air-dried, and metaphase spreads were visualized using Vectashield Mounting Medium containing DAPI (REACTOLAB S.A., Servion, Switzerland). Microscopic images were acquired using an Axio Imager.Z2 microscope (Carl Zeiss AG, Feldbach, Switzerland) and analyzed with Isis software (MetaSystems Hard & Software GmbH, Altlussheim, Germany).

**Bulk RNA sequencing**

Total RNA was extracted using the RNeasy Kit (QIAGEN, Cat. No. 74104), or Arcturus PicoPure RNA Isolation Kit (Applied Biosystems, Cat. No. KIT0204) following the manufacturer’s protocols. High-throughput next-generation sequencing was performed at the Functional Genomics Center Zurich on a NovaSeq 6000 Sequencing System (Illumina®, San Diego, CA, USA) for sequencing of ER-HoxB8-tetCre-*Trp53*-R245W-GFP cells after etoposide challenge or an Illumina NovaSeq X Plus (Illumina®, San Diego, CA, USA) for sequencing of ER-HoxB8-tetCre-*Trp53*-R245W-GFP cells with MDM2 overexpression, as well as for sequencing of primary murine LSK cells and leukemias. Quality control, mapping and differential gene expression analysis was performed using the SUSHI bioinformatics pipeline. Gene set enrichment analysis (GSEA) was performed using the GSEA software jointly developed by UC San Diego and the Broad Institute (RRID:SCR_003199, Subramanian et al, 2005). Volcano plots and heatmaps were visualized using RStudio (RRID:SCR_000432) packages “EnhancedVolcano” (RRID:SCR_018931) and “pheatmap” (RRID:SCR_016418) running R (RRID:SCR_001905, version 4.3.1).

**Whole genome sequencing of ER-HoxB8-tetCre-*Trp53*-fl-R245W-GFP single cell colonies**

For whole genome sequencing of ER-HoxB8-tetCre-*Trp53*-fl-R245W-GFP single cell colonies, 1.5 x 10⁶ cells of the indicated genotypes were treated with 40 μM etoposide for 1h. Cells were washed twice with PBS and incubated in fresh, drug-free culture medium until recovery of at least 1.5 x 10⁶ viable cells. Subsequently, treatment was repeated for two additional cycles. After that, single cell sorting into 96-well plates was performed using an Aria III 4L (Becton Dickinson, Franklin Lakes, NJ, USA). Colonies were allowed to grow until they reached 1 x 10⁶ viable cells, after which DNA was isolated using the QIAamp DNA Mini Kit (Qiagen). High-throughput next-generation sequencing was performed at the Functional Genomics Center Zurich on a NovaSeq 6000 Sequencing System (coverage > 10x; paired end 150 bp; library protocol: Illumina Truseq Nano (Illumina®, San Diego, CA, USA)). Mapping was done using the Burrows-Wheeler Aligner (BWA, RRID:SCR_010910) with the mm39 reference. For SNV analysis, the Octopus basic model with default settings was used, looking at germline variants. CNV analysis was performed using the control freec C++ tool (RRID:SCR_010822) (window = 50000; reference = mm39), without looking at allelic content.

**Whole genome sequencing of murine AMLs**

Whole bone marrow cells of AML-bearing mice frozen at -80°C were thawed, stained and sorted by FACS for live/CD45-/GFP+/lineage-/c-Kit+ cells, identifying AML blasts. DNA was isolated using the QIAamp DNA Mini Kit (Qiagen). High-throughput next-generation sequencing was performed at the Functional Genomics Center Zurich on a NovaSeq X Plus (coverage 30x; paired end 150 bp; library protocol: Illumina Truseq Nano (Illumina®, San Diego, CA, USA)). Mapping was done using the Burrows-Wheeler Aligner (BWA) using sequencing data from bone marrow cells of healthy *Trp53*-fl-R245W-GFP;SCL-CreERT mice as reference. CNV analysis was performed according the MoCaSeq CNV workflow for WGS (3). First, bam files from control and AML mice were subjected to the readCounter program from the HMMcopy Utils repository to generate the read counts in windows non-overlapping windows of fixed width across the genome. Subsequently, these counts were corrected for mappability and GC content. Regions with increased variability obtained from public resources were excluded from the analysis. Next, log2ratio of counts between AML and control was calculated per sample. Segmentation was performed using the DNAcopy R package. Log2ratio was converted to absolute copy number and used for generating the heat map of CNVs across the AML cohort. All the technical details and the code to run the analysis are included in (3) and the GitHub repository https://github.com/roland-rad-lab/MoCaSeq.

***Trp53*-fl-R245W-GFP genotyping/recombination PCR**

Bone marrow cells from AML-bearing mice were subjected to FACS to isolate a pure population of AML blasts for assessing loss of heterozygosity at the *Trp53* locus. Similarly, bone marrow cells from healthy, tamoxifen-induced mice were sorted into GFP-negative, GFP-low, and GFP-high populations to evaluate Cre-mediated recombination of transgenic alleles. DNA was subsequently extracted using the QIAamp DNA Mini Kit (Qiagen). PCR amplification was conducted with the KAPA Taq ReadyMix (Roche, Cat. No. KK1006) using 10 ng of DNA input and specific primers (5`-CCAGCTGCTAGAGACAGTTGAGG-3`; 5`-AGCCAGCAAAGAGAGGACTGG-3`). PCR products were resolved on a 1.5% agarose gel containing 1:10,000 GelRed and electrophoresed at 120V for 1 hour. Gels were visualized using GelStick Imager (Intas).

**Amplicon sequencing**

Genomic DNA was extracted from FACS-sorted GFP^neg^, GFP^low^, and GFP^high^ LSK cells isolated from mice with biallelic *Trp53*-fl-R245W-GFP alleles. Target regions were amplified using Q5 High-Fidelity 2X Master Mix (New England Biolabs, Cat. No. M0492L) and specific primers (forward: 5′-CCAGCTGCTAGAGACAGTTGAGG-3′; reverse: 5′-AGCCAGCAAAGAGAGGACTGG-3′). Amplicon sequencing was performed by the MGH CCIB DNA Core Facility (Cambridge, MA) using their Complete Amplicon Sequencing workflow. PCR amplicons (>400 bp) were enzymatically fragmented, processed through standard next-generation sequencing library preparation, and subjected to massively parallel sequencing followed by automated *de novo* assembly. Relative frequencies of wild-type (non-recombined, FRT-containing) and mutant (recombined, FRT-excised) reads were quantified and plotted.

**Mouse experiments**

*Trp53*-fl-R245W-GFP mice (4) and SCL-CreERTmice (5) were used in this study, along with C57BL/6 (RRID:IMSR_JAX:000664) and C57BL/6-Ly5.1 (RRID:IMSR_JAX:002014) wild-type mice obtained from Charles River. *Trp53*-fl-R245W-GFP;SCL-CreERT mice were bred to generate animals homozygous or heterozygous for the *Trp53*-fl-R245W-GFP allele and hemizygous for the SCL-CreERT allele. All mice were maintained under specific pathogen-free conditions in accordance with the guidelines of the Swiss Federal Veterinary Office. Experiments were approved by the Veterinary Office of the Canton of Zurich (License ZH181/2021).

To induce Cre-mediated recombination of the *Trp53* locus, 6-12-week-old female *Trp53*-fl-R245W-GFP;SCL-CreERT mice were injected intraperitoneally with 100 mg/kg tamoxifen (Sigma Aldrich, Cat. No. T5648), dissolved in ethanol and diluted in corn oil (Sigma Aldrich, Cat. No. C8267) for 1-5 consecutive days. Peripheral blood was collected via tail vein puncture at specified time points, with at least two weeks between collections. For studies investigating clonal expansion and malignant transformation of *Trp53* mutant cells, mice were randomly divided into two groups five weeks post tamoxifen induction. One group was subjected to two doses of sublethal γ-irradiation (475 cGy, RS-2000 irradiator (Rad Source, Buford, GA, USA)) with a four week interval, while the other remained non-irradiated. Mice were sacrificed either at predetermined endpoints or upon reaching humane endpoint criteria. Termination criteria were scored by an experimenter blinded to the experimental cohort.

In transplantation experiments, 12-week-old female C57BL/6 recipient mice received myeloablative irradiation (2 × 475 cGy with a 4-hour interval) before tail vein injection of 2 x 10⁶ thawed whole bone marrow cells from AML-bearing donor mice, combined with 0.5 x 10⁶ whole bone marrow cells from C57BL/6-Ly5.1 mice. Recipient mice were sacrificed upon meeting humane endpoint criteria.

Random allocation of mice to experimental groups was performed by an independent individual, taking into account balanced age distribution across groups; however, no formal randomization method was applied. Sample size was determined empirically based on prior experience with comparable *in vivo* experiments aiming to provide sufficient power for statistical analyses. The exact number of animals used in each experiment is specified in the corresponding figure legends. Mice that died within the first 14 days following γ-irradiation were excluded from analysis, as these cases reflected acute irradiation toxicity rather than experimental outcome. Blinding could not be implemented during the γ-irradiation procedures due to the technical nature of the interventions. Following irradiation, mice were assigned randomized identification codes to ensure unbiased assessment of endpoint criteria during subsequent monitoring and analysis.

***Ex vivo* processing of murine samples**

At the termination time point, spleens and tumors were excised, blood was collected via heart puncture, and bone marrow was harvested by flushing femora, tibiae, and pelvic bones with FACS buffer. Single-cell suspensions from the organs were prepared by passing them sequentially through 0.7 µm and 0.45 µm filters into FACS buffer. Red blood cells were lysed by incubating the cells with RBC lysis buffer (Biolegend, Cat. No. 420301) for 10 minutes. Cells were either frozen at -80°C in FACS buffer containing 50% FBS and 10% DMSO or directly processed for flow cytometric analysis. Spleen weights were recorded using a AE163 precision scale (Mettler Toledo). Femora were fixed in 4% PFA (Paraformaldehyde solution 4% in PBS, Santa Cruz Biotechnology, Cat. No. sc-281692) for subsequent histological analysis. Blood samples were analyzed for complete blood counts using the ADVIA 2120i Hematology system.

**Statistical analyses**

Statistical significance was assessed using one-way ANOVA followed by Tukey’s multiple comparisons post hoc test, unpaired two-tailed t-test, or log-rank (Mantel–Cox) test, as indicated in the figure legends. Analyses were performed using GraphPad Prism software (version 10.2.1; RRID:SCR_002798; GraphPad Software, Boston, MA, USA). Data distribution and variance were assessed to confirm that test assumptions were met. Variation within each group is shown as standard error of the mean (SEM).

**SUPPLEMENTARY TABLES**

**Table 1: Antibody list**

| Target | Conjugation | Clone | Dilution factor |
| --- | --- | --- | --- |
| Mouse CD45 | PerCP/Cyanine5.5 | 30-F11 (Biolegend) | 1:100 |
| Mouse CD3e | PE | 145-2C11 (eBioscience) | 1:100 |
| Mouse CD19 | PE-Cyanine7 | eBio1D3 (eBioscience) | 1:50 |
| Mouse CD11b | APC-eFluor 780 | M1/70 (eBioscience) | 1:500 |
| Mouse CD11b | PE-Cyanine5 | M1/70 (eBioscience) | 1:500 |
| Mouse F4/80 | APC-eFluor 780 | BM8 (Invitrogen) | 1:100 |
| Mouse Ly-6G/Ly-6C | APC | RB6-8C5 (eBioscience) | 1:100 |
| Mouse CD8a | PE-Cyanine5 | 53-6.7 (eBioscience) | 1:100 |
| Mouse CD4 | APC | GK1.5 (eBioscience) | 1:100 |
| Mouse CD45R (B220)* | Biotin | RA3-6B2 (eBioscience) | 1:500 |
| Mouse CD11b* | Biotin | M1/70 (eBioscience) | 1:500 |
| Mouse CD3e* | Biotin | 145-2C11 (eBioscience) | 1:500 |
| Mouse CD8a* | Biotin | 53-6.7 (eBioscience) | 1:500 |
| Mouse Ly-6G/Ly-6C* | Biotin | RB6-8C5 (eBioscience) | 1:500 |
| Mouse CD4* | Biotin | GK1.5 (eBioscience) | 1:500 |
| Mouse Ter119* | Biotin | TER-119 (Biolegend) | 1:500 |
| Mouse NK1.1* | Biotin | PK136 (eBioscience) | 1:500 |
| Biotin | Qdot 605 Streptavidin | Invitrogen | 1:250 |
| Mouse CD117 (c-Kit) | PE-Cyanine7 | 2B8 (eBioscience) | 1:200 |
| Mouse Ly-6A/E (Sca-1) | APC-Cyanine7 | D7 (Biolegend) | 1:100 |
| Mouse CD16/32 | Brilliant Violet 711 | 93 (Biolegend) | 1:50 |
| Mouse CD34 | eFluor 660 | RAM34 (eBioscience) | 1:50 |
| Mouse CD127 | PE-Cyanine5 | A7R34 (eBioscience) | 1:50 |
| Mouse CD71 | PE | RI7217 (Biolegend) | 1:100 |
| Mouse CD135 | PerCP-eFluor 710 | A2F10 (Invitrogen) | 1:50 |
| Mouse CD48 | Alexa Fluor 700 | HM48-1 | 1:100 |
| Mouse CD150 | Brilliant Violet 785 | TC15-12F12.2 (Biolegend) | 1:50 |
| Phospho-Histone H2A.X | Biotin | JBW301 (Sigma-Aldrich) | 1:250 |
| Biotin | APC Streptavidin | Biolegend | 1:333 |

* used for lineage antibody-cocktail

**REFERENCES**

1. Fischer M. Conservation and divergence of the p53 gene regulatory network between mice and humans. Oncogene. 2019;38(21):4095-109.

2. Wang GG, Calvo KR, Pasillas MP, Sykes DB, Hacker H, Kamps MP. Quantitative production of macrophages or neutrophils ex vivo using conditional Hoxb8. Nat Methods. 2006;3(4):287-93.

3. Lange S, Engleitner T, Mueller S, Maresch R, Zwiebel M, Gonzalez-Silva L, et al. Analysis pipelines for cancer genome sequencing in mice. Nat Protoc. 2020;15(2):266-315.

4. Murai K, Skrupskelyte G, Piedrafita G, Hall M, Kostiou V, Ong SH, et al. Epidermal Tissue Adapts to Restrain Progenitors Carrying Clonal p53 Mutations. Cell Stem Cell. 2018;23(5):687-99 e8.

5. Gothert JR, Gustin SE, Hall MA, Green AR, Gottgens B, Izon DJ, et al. In vivo fate-tracing studies using the Scl stem cell enhancer: embryonic hematopoietic stem cells significantly contribute to adult hematopoiesis. Blood. 2005;105(7):2724-32.
